# SARS-CoV-2 non-structural protein 6 triggers NLRP3-dependent pyroptosis by targeting ATP6AP1

**DOI:** 10.1038/s41418-021-00916-7

**Published:** 2022-01-08

**Authors:** Xiao Sun, Yingzhi Liu, Ziheng Huang, Wenye Xu, Wei Hu, Lina Yi, Zhe Liu, Hung Chan, Judeng Zeng, Xiaodong Liu, Huarong Chen, Jun Yu, Francis Ka Leung Chan, Siew Chien Ng, Sunny Hei Wong, Maggie Haitian Wang, Tony Gin, Gavin Matthew Joynt, David Shu Cheong Hui, Xuan Zou, Yuelong Shu, Christopher Hon Ki Cheng, Shisong Fang, Huanle Luo, Jing Lu, Matthew Tak Vai Chan, Lin Zhang, William Ka Kei Wu

**Affiliations:** 1 School of Biomedical Sciences, The Chinese University of Hong Kong, Hong Kong, China; 2 Department of Anaesthesia and Intensive Care and Peter Hung Pain Research Institute, The Chinese University of Hong Kong, Hong Kong, China; 3 Department of Medicine and Therapeutics, The Chinese University of Hong Kong, Hong Kong, China; 4 Department of Gastroenterology, Shenzhen Hospital, Southern Medical University, Shenzhen, Guangdong China; 5 Guangdong Provincial Institution of Public Health and Guangdong Provincial Center for Disease Control and Prevention, Guangdong, China; 6 State Key Laboratory of Digestive Diseases, Li Ka Shing Institute of Health Sciences, The Chinese University of Hong Kong, Hong Kong, China; 7 Institute of Digestive Disease, The Chinese University of Hong Kong, Hong Kong, China; 8 CUHK Shenzhen Research Institute, Shenzhen, China; 9 Lee Kong Chian School of Medicine, Nanyang Technological University, Singapore, Singapore; 10 The Jockey Club School of Public Health and Primary Care, The Chinese University of Hong Kong, Hong Kong, China; 11 Shenzhen Center for Disease Control and Prevention, Shenzhen, Guangdong China; 12 School of Public Health (Shenzhen), Sun Yat-sen University, Shenzhen, Guangdong China

**Keywords:** Cell death and immune response, Microbiology

## Abstract

A recent mutation analysis suggested that Non-Structural Protein 6 (NSP6) of the Severe Acute Respiratory Syndrome Coronavirus 2 (SARS-CoV-2) is a key determinant of the viral pathogenicity. Here, by transcriptome analysis, we demonstrated that the inflammasome-related NOD-like receptor signaling was activated in SARS-CoV-2-infected lung epithelial cells and Coronavirus Disease 2019 (COVID-19) patients’ lung tissues. The induction of inflammasomes/pyroptosis in patients with severe COVID-19 was confirmed by serological markers. Overexpression of NSP6 triggered NLRP3/ASC-dependent caspase-1 activation, interleukin-1β/18 maturation, and pyroptosis of lung epithelial cells. Upstream, NSP6 impaired lysosome acidification to inhibit autophagic flux, whose restoration by 1α,25-dihydroxyvitamin D_3_, metformin or polydatin abrogated NSP6-induced pyroptosis. NSP6 directly interacted with ATP6AP1, a vacuolar ATPase proton pump component, and inhibited its cleavage-mediated activation. L37F NSP6 variant, which was associated with asymptomatic COVID-19, exhibited reduced binding to ATP6AP1 and weakened ability to impair lysosome acidification to induce pyroptosis. Consistently, infection of cultured lung epithelial cells with live SARS-CoV-2 resulted in autophagic flux stagnation, inflammasome activation, and pyroptosis. Overall, this work supports that NSP6 of SARS-CoV-2 could induce inflammatory cell death in lung epithelial cells, through which pharmacological rectification of autophagic flux might be therapeutically exploited.

## Introduction

The Severe Acute Respiratory Syndrome Coronavirus 2 (SARS-CoV-2) is a zoonotic, novel coronavirus that emerged at the end of 2019, causing Coronavirus Disease 2019 (COVID-19) [1]. Although as high as 81% of patients positive for SARS-CoV-2 are asymptomatic [2], a substantial proportion of patients with COVID-19 develop pneumonia and may progress to acute respiratory distress syndrome which is characterized by a rapid onset of widespread inflammation in the lungs and is associated with a significant mortality [3]. At present, our understanding of the molecular mechanisms underlying SARS-CoV-2-driven deranged immune responses and hyperinflammatory state remains largely fragmented. To this end, aberrant activation of inflammasomes might play a role, and it has recently been shown that the serum levels of inflammasome-derived products, namely active caspase-1 (the p20 subunit) and interleukin (IL)-18, correlate with poor clinical outcomes in patients with COVID-19 [4].

Autophagy functions as an important host defense mechanism to counteract viral infection. This lysosome-dependent degradation process not only selects viral components for lysis, but also facilitates antigen processing and adaptive immune responses [5]. However, positive-strand RNA viruses, including coronaviruses, have evolved strategies to hijack this host machinery for their intracellular replication and propagation [6]. For example, different proteins encoded by SARS-CoV, the causative agent of SARS, have been shown to impair lysosomal function and autophagic flux in the airway epithelium [7–9]. Notably, among 16 non-structure protein (NSPs) of coronavirus, NSP6 has been shown to induce autophagosome formation from host endoplasmic reticulum [10]. Intriguingly, the NSP6 ortholog of Infectious Bronchitis Virus, an avian coronavirus, impaired starvation-induced autophagosome and lysosome expansion [7], suggesting the complex regulation of autophagy by NSP6. Pertinent to the pathogenicity of SARS-CoV-2, NSP6 L37F mutation has recently been linked to viral hypotoxicity [11].

Since autophagic flux impairment is known to activate NLRP3 inflammasomes to mediate the maturation of pro-inflammatory cytokines IL-1β and IL-18 [5], we postulated that SARS-CoV-2 may encode virulence factor(s) that activates this inflammatory cascade and leads to severe disease manifestation in COVID-19 patients. A recent study has already shown that the SARS-CoV-2 protein ORF3a inhibits autophagosome-lysosome fusion by interacting with VPS39 [12], a component of the homotypic fusion and protein sorting (HOPS) complex. In this study, we show that NSP6 is another SARS-CoV-2 protein that inhibits the lysosome-autophagy system. Such action is mediated by the direct interaction of NSP6 with a lysosomal proton pump component and this instigates the downstream NLRP3 inflammasome-dependent pyroptotic pathway in lung epithelial cells.

## Results

### Transcriptome analysis revealed upregulation of inflammasome-related NOD-like receptor signaling in SARS-CoV-2-infected lung epithelial cells

**Fig. 1:**
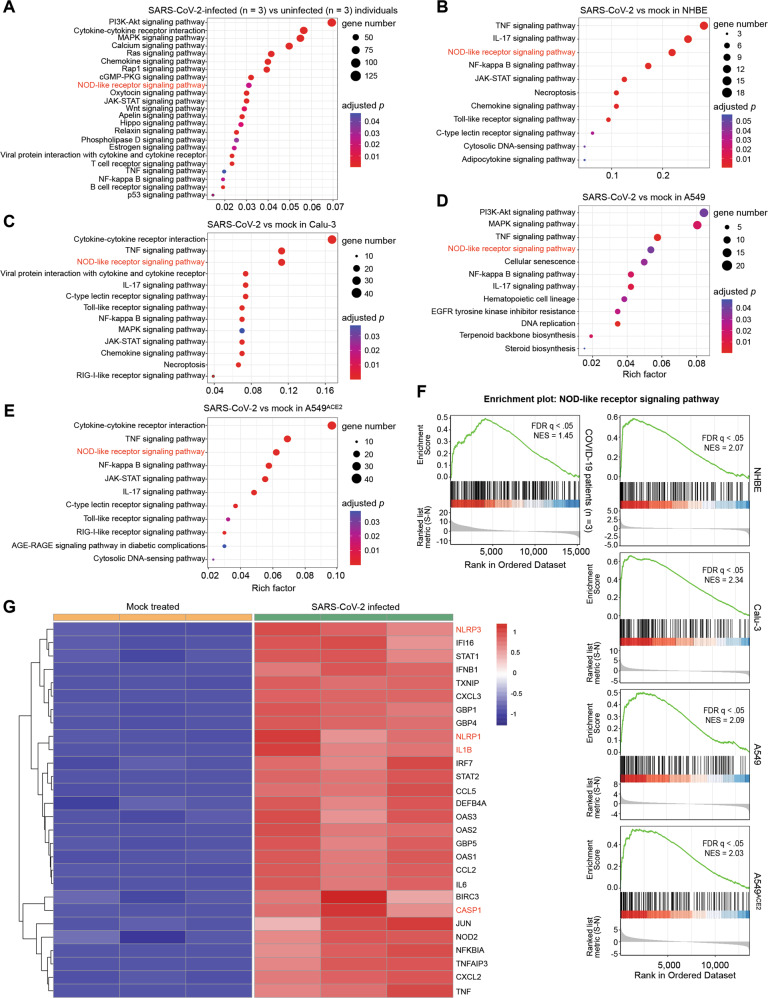
SARS-CoV-2 induced expression of genes enriched in the inflammasome-related NOD-like receptor signaling pathway in human airway epithelial cells. **A–E** Dot plot visualization of KEGG pathway enrichment. Pathway enrichment analyses were performed using R package clusterProfiler. The color of the dots represents the adjusted *p* value for each enriched KEGG pathway, and the size of the dots represents the count of genes enriched in the corresponding pathway. **F** Gene set enrichment analysis (GSEA) enrichment plot of the NOD-like receptor signaling pathway. **G** Heatmap of differentially expressed genes (DEGs) in NOD-like receptor signaling pathway. The included genes have the absolute values of log_2_ (fold change) more than 1 and adjusted *p* value less than 0.05.

We first performed Kyoto encyclopedia of genes and genomes (KEGG) pathway enrichment analysis of published transcriptomes of COVID-19 patients’ lung tissues and SARS-CoV-2-infected lung epithelial cell lines to examine the possible activation of the inflammasome pathway during COVID-19 pathogenesis. As shown in Fig. 1A, differentially expressed genes between lung samples derived from three COVID-19 patients with high viral load and human lung biopsies derived from three uninfected individuals as control showed a significant enrichment in the inflammasome-related NOD-like receptor signaling pathway. Similarly, genes differentially expressed upon in vitro SARS-CoV-2 infection in airway epithelial cell lines (NHBE, Calu-3, and A549 without or with ACE2 overexpression) were enriched in the NOD-like receptor signaling pathway (Fig. 1B–E). The activation of NOD-like receptor signaling in SARS-CoV-2-infected lung biopsies and epithelial cell lines was further confirmed by gene set enrichment analysis (GSEA; all *q* < 0.05) (Fig. 1F). Heatmap visualizing differentially expressed genes in NOD-like receptor signaling revealed the pronounced upregulation of genes encoding IL-1β and the inflammasome components NLRP3, NLRP1 and CASP1 (Fig. 1G). These findings confirmed upregulation of the inflammasomes at the transcriptomic level in lung epithelial cells as a host response to SARS-CoV-2 infection.

### SARS-CoV-2 triggered IL-1β, IL-18 and M65 release in COVID-19 patients

**Fig. 2:**
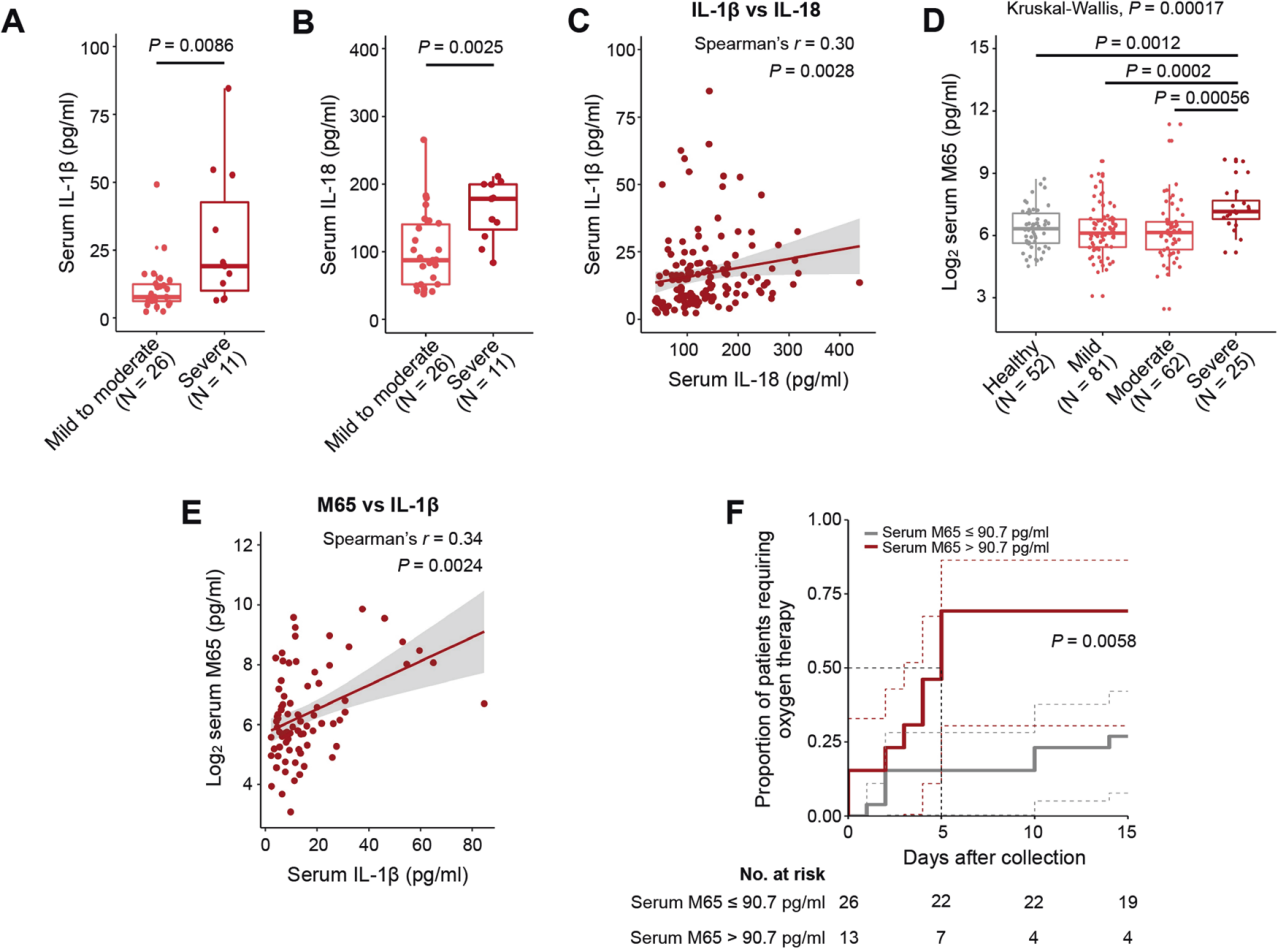
SARS-CoV-2 triggered IL-1β, IL-18 and M65 release in COVID-19 patients. Quantification of serum IL-1β (**A**) and IL-18 (**B**) levels in COVID-19 patients with mild-to-moderate and severe disease. **C** Correlation between serum levels of IL-1β and IL-18 (*n* = 91). **D** Log_2_ serum M65 levels in heathy subjects and COVID-19 patients with different disease severity. **E** Correlation between serum log_2_ M65 (*n* = 78) and IL-1β. **F** Kaplan–Meier estimate of the requirement of the oxygen therapy by serum M65 levels. Dashed lines indicate 95% confidence intervals. Significance was assessed using Wilcoxon log-rank (**A**, **B**) or Kruskal–Wallis with Wilcox log-rank test (**D**). Significance and correlation coefficient were assessed using Spearman’s correlation test with 95% confidence interval (**C**, **E**). Box plots with outliners and points representing individual sample were shown.

To verify our findings from the transcriptome analysis, a total of 220 serum samples were collected from 157 COVID-19 patients and 52 close contacts without SARS-CoV-2 infection confirmed by a negative SARS-CoV-2 PCR test. Only a subset of 91 specimens had measurements of circulating IL-1β and IL-18 levels. Clinical characteristics of this cohort and part of the results on IL-1β and IL-18 have been previously reported [13, 14]. We found that serum concentrations of IL-1β (*p* = 0.0086) and IL-18 (*p* = 0.0025) were significantly higher in patients with severe COVID-19 as compared with those suffering from mild-to-moderate COVID-19 (Fig. 2A, B). Serum IL-1β and IL-18 levels were also positively correlated with each other in patients with COVID-19 (Spearman’s *r* = 0.30, *p* = 0.0028) (Fig. 2C). An epithelial cell death marker, M65 [15], was further measured in all 220 specimens. Serum concentrations (log_2_) of M65 were higher among patients with severe COVID-19 (Fig. 2D) and positively correlated with serum IL-1β concentrations (Spearman’s *r* = 0.34, *p* = 0.0024) (Fig. 2E). These serological data suggested the activation of inflammasomes/pyroptosis in patients with severe COVID-19. Importantly, in a subset of 39 COVID-19 patients (Supplementary Fig. S1 for patients’ disease course) whose specimens were collected within a week of hospital admission and before clinical deterioration, high M65 serum concentration (>90.7 pg/ml) was found to significantly predict the requirement of oxygen therapy (*p* = 0.0058; Fig. 2F for the Kaplan–Meier curve) independent of age and sex (Table 1 for univariate and multivariate Cox regression analyses).

**Table 1:** Cox regression models using M65, age and sex for predicting oxygen therapy outcome.

	Never used O_2_	Required O_2_	Uni-variate		Multi-variate	
	*N* = 23	*N* = 16	HR (95% CI)	*p* value	HR (95% CI)	*p* value
M65 (Median, IQR)	46.4 (28.3–80.8)	96.6 (49.0–123.3)	1.0 (0.99–1.01)	0.140		
M65 > 90.7 pg/mL (*N* (%))	4 (17.4)	9 (56.3)	3.8 (1.4–10.3)	**0.009**	3.6 (1.2–10.5)	**0.018**
Age (Median, IQR)	39.0 (28.5–49.5)	48.5 (36.5–57.8)	1.0 (0.99–1.06)	0.141	1.0 (0.98–1.0)	0.352
Female (N (%))	11 (47.8)	8 (50.0)	0.9 (0.3–2.5)	0.886	1.3 (0.5–3.5)	0.654

*HR* hazzard ratio, *CI* confidence intervals.

### Enforced expression of SARS-CoV-2 NSP6 induced inflammasome activation in lung epithelial cells

**Fig. 3:**
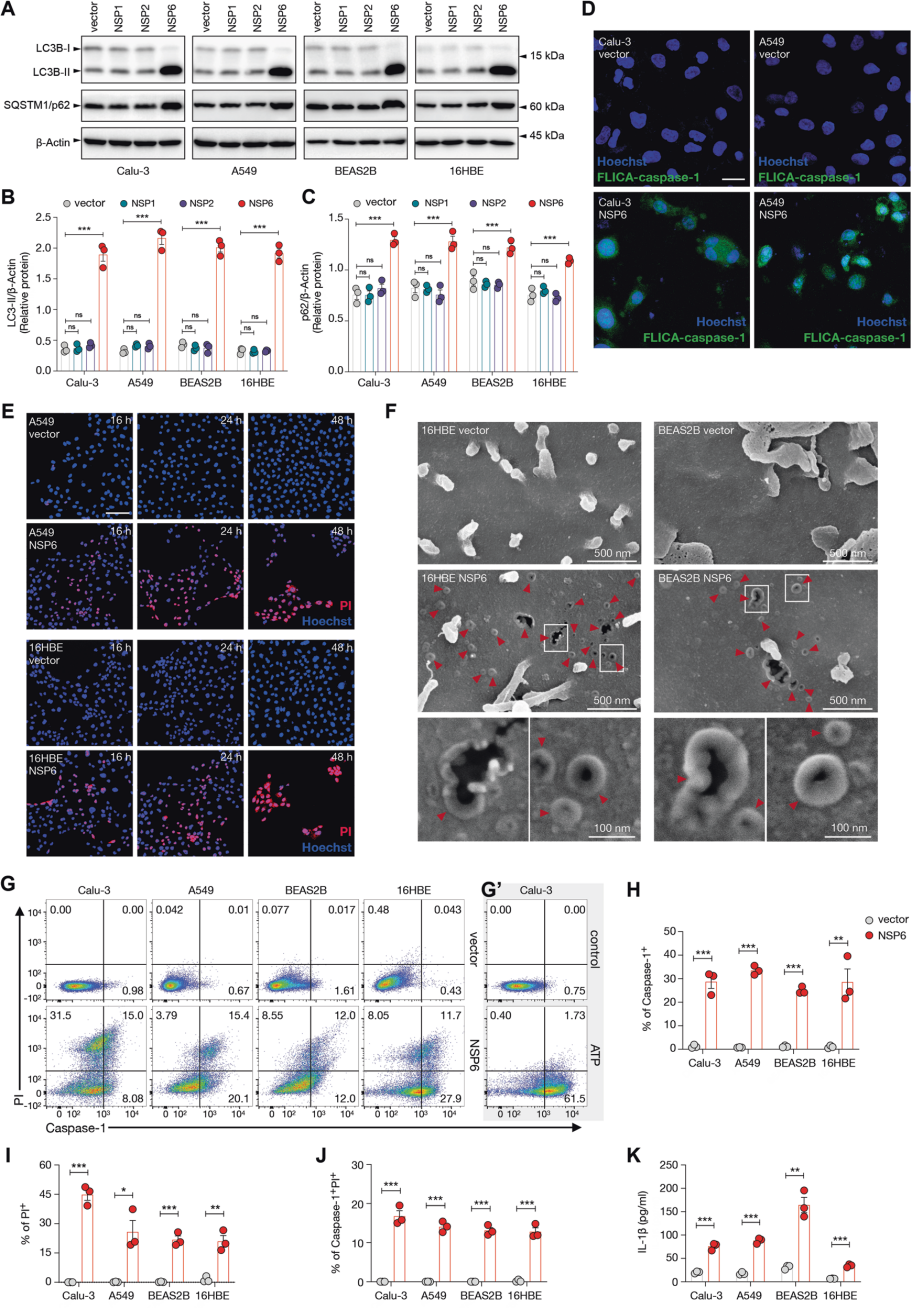
NSP6 impaired the autophagic flux and induced the activation of inflammasomes in lung epithelial cells. **A**, **B** Calu-3, A549, BEAS2B and 16HBE cells were transfected with control empty vector or NSP1-, NSP2- or NSP6-encoding plasmid for 24 h. **A** Whole-cell lysates were examined for LC3B and SQSTM1/p62 expression. Quantification of relative protein levels is displayed as the ratio of intensity of LC3B-II (**B**) or SQSTM1/p62 (**C**) to β-actin. **D** Calu-3 and A549 cells were transfected with control empty vector or NSP6-encoding plasmid for 24 h. Active caspase-1 was visualized using fluorescently-labelled inhibitor of caspase-1 (FLICA) probe (green). Nuclei were visualized with Hoechst stain (blue). Scale bar 20 μm. **E** A549 and 16HBE cells were transfected with control empty vector or NSP6-encoding plasmid for 16, 24 or 48 h. Cell death and membrane rupture were identified using propidium iodide (PI) staining. Nuclei were visualized with Hoechst stain (blue). Scale bar 100 μm. **F** 16HBE and BEAS2B cells were transfected with control empty vector or NSP6-encoding plasmid for 24 h. Representative scanning electron microscopy images of top-down views were shown. GSDMD-NT pores are indicated by red arrows. **G**–**K** Calu-3, A549, BEAS2B and 16HBE cells were transfected with control empty vector or NSP6-encoding plasmid for 24 h. **G** Cell death was monitored by PI staining and active caspase-1 was identified by FLICA probe. Fluorescence intensity was analyzed by flow cytometry. **G*’*** Calu-3 cells treated with ATP (2 mM, 2 h) was used as a positive control. Quantification of caspase-1^+^ (**H**), PI^+^ (**I**) and caspase-1^+^PI^+^ (**J**) cells are displayed as percentages. **K** Quantification of IL-1β secretion in cell culture supernatant showed increased IL-1β levels upon NSP6 overexpression. β-Actin was used as a loading control. Significance was assessed using one-way ANOVA with Dunnett’s multiple comparison test (**B**, **C**) or unpaired two-tailed *t*-test (**H–K**). All quantitative data are presented as means ± SD. from three independent experiments. *p < 0.05; **p < 0.01; ***p < 0.001; ns no significance.

To determine if NSP6 of SARS-CoV-2 impairs the autophagic flux to activate inflammasomes, NSP6-encoding plasmid was transfected into four human airway epithelial cell lines (Calu-3, A549, BEAS2B and 16HBE). Effects of overexpression of two other SARS-CoV-2 NSPs, namely NSP1 and NSP2, on autophagic flux were also explored. As compared with the control plasmid, enforced expression of NSP6 but not NSP1 or NSP2 induced the concomitant accumulation of LC3B-II and SQSTM1/p62 in all four cell lines (Fig. 3A–C), which was suggestive of the impairment of autophagic flux [16]. The expression level of NSP6 as compared with other SARS-CoV-2-encoded genes in lung tissues from COVID-19 patients (GSE163959) was shown in Supplementary Fig. S2.

A fluorescently labelled inhibitor of caspase-1 (FLICA) probe was then used to visualize the activation of caspase-1 [17], an essential component of the inflammasome (Fig. 3D). NSP6-induced membrane rupture was further visualized by propidium iodide uptake (Fig. 3E) and the formation of p30 N-terminal fragment of gasdermin D (GSDMD-NT) pore on the cell surface, which is key feature of inflammasome-mediated pyroptosis (Fig. 3F). The proportion of FLICA-positive cells was substantially increased upon NSP6 overexpression, indicating caspase-1 activation (Fig. 3G). Flow cytometry of FLICA/propidium iodide double-stained cells not only confirmed the strong activation of caspase-1 by NSP6, but also showed that a significant proportion of active caspase-1-positive cells had lost the membrane integrity (Fig. 3H–J). These features were consistent with the induction of pyroptosis, a highly inflammatory form of cell death [18]. Concordantly, NSP6 overexpression induced the release of IL-1β into the supernatants (Fig. 3I).

### NSP6 induced the accumulation of non-digestive autophagosomes without altering autophagosome-lysosome fusion

**Fig. 4:**
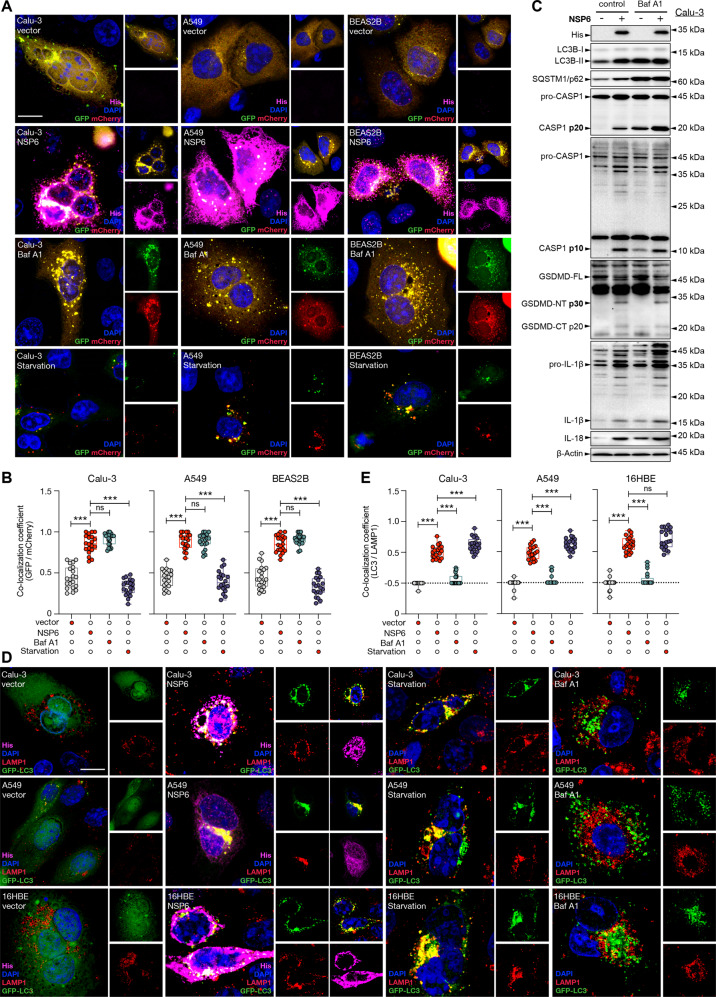
NSP6 induced accumulation of non-digestive autophagosomes without inhibiting autophagosome-lysosome fusion. **A**, **B** Calu-3, A549 and BEAS2B cells were co-transfected with mCherry-GFP-LC3 plasmid and control empty vector or NSP6-encoding plasmid for 24 h. Bafilomycin A1 (Baf A1; 100 nM, 24 h) and starvation (no serum, 24 h) were used as positive and negative control, respectively. **A** Representative fluorescence images of mCherry-GFP-LC3 fusion protein (GFP, green; mCherry, red) and his-tagged NSP6 (magenta) expression. Nuclei were stained with DAPI (blue). Scale bar 10 μm. **B** Quantification of colocalization coefficient is displayed as the percentage of punctate mCherry signals that were positive for GFP signals. Twenty cells from each group were randomly selected for statistical analysis. **C** Calu-3 cells were transfected with control empty vector or NSP6-encoding plasmid for 24 h. Baf A1 was added to culture 1 h after transfection until cells were harvested. Whole-cell lysates were examined for his-tagged NSP6, LC3B, SQSTM1/p62, IL-18, inactive/full-length and active forms of CASP1 (p20 and p10), GSDMD (p30 NT fragment) and IL-1β (p17). **D**, **E** Calu-3, A549 and 16HBE cells were co-transfected with GFP-LC3 plasmid and control empty vector or NSP6-encoding plasmid for 24 h. Starvation and Baf A1 were used as positive and negative control, respectively. **D** Representative fluorescence images of GFP-LC3 (green), LAMP1 (red) and his-tagged NSP6 (magenta) expression. Nuclei were stained with DAPI (blue). Scale bar 10 μm. **E** Quantification of colocalization coefficient is displayed as the percentage of punctate LAMP1 signals that were positive for LC3B signals. Twenty cells from each group were randomly selected for statistical analysis. Significance was assessed using one-way ANOVA with Tukey’s multiple comparison test. All quantitative data are presented as means ± SD. ***p < 0.001; ns no significance.

As a potent suppressor of inflammation, autophagy clears damaged mitochondria that release reactive oxygen species (ROS) and mitochondrial DNA, thereby suppressing inflammasome activation [19]. Since Western blot results revealed that NSP6 might impair autophagic flux at the late stage (i.e. impeding autophagosome-lysosome fusion or attenuating lysosome-dependent degradation), we transfected the cells with plasmid encoding a mCherry-GFP-LC3B fusion protein. We observed that enforced expression of NSP6 led to the accumulation of non-digestive autophagosomes (mCherry^+^, GFP^+^ puncta) in Calu-3, A549 and BEAS2B cells. Bafilomycin A1, a potent inhibitor of vacuolar type H^+^-ATPase, was used as a positive control (Fig. 4A, B). Similar to the action of NSP6, bafilomycin A1 increased the protein levels of LC3B‐II, SQSTM1/p62, p20 and p10 active subunits of caspase-1, p30 NT fragment of GSDMD, and mature IL-1β and IL-18 (Fig. 4C), confirming that autophagy is upstream to inflammasomes in lung epithelial cells. However, unlike bafilomycin A1 that not only disrupts lysosome acidification but also inhibits autophagosome-lysosome fusion [20], NSP6 did not have significant impact on the latter as visualized by the colocalization of LC3B with lysosome-associated membrane protein 1 (LAMP1) (Fig. 4D, E).

### NSP6 inhibited lysosome acidification by targeting ATP6AP1

**Fig. 5:**
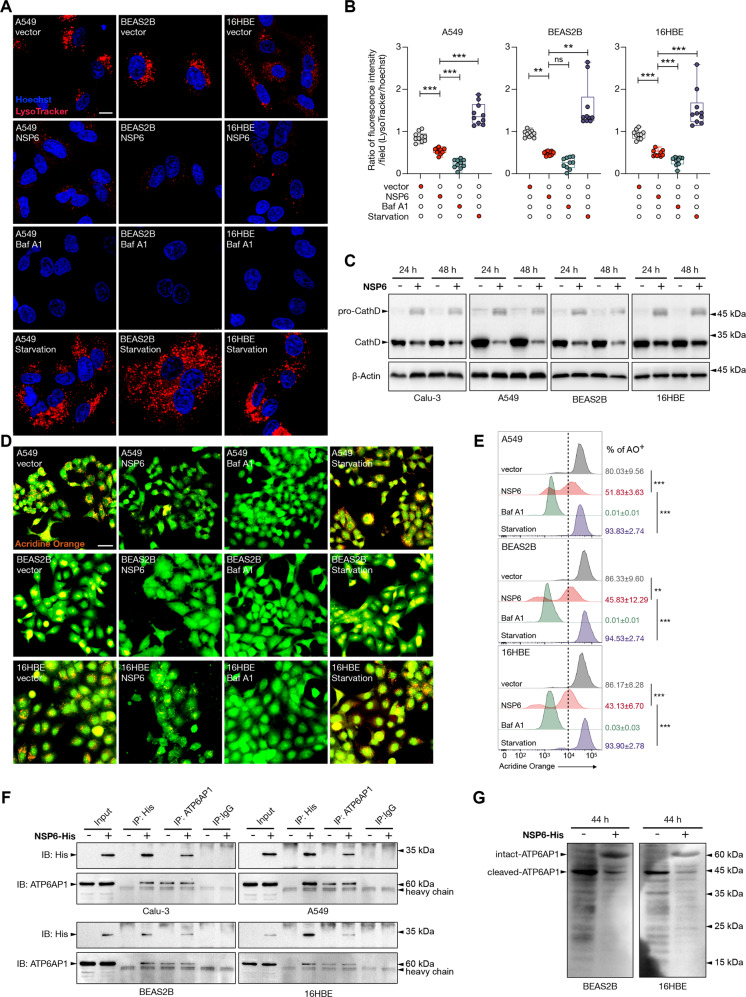
NSP6 suppressed lysosome acidification and interacted with ATP6AP1. **A**, **B** A549, BEAS2B and 16HBE cells were transfected with control empty vector or NSP6-encoding plasmid for 24 h then incubated with LysoTracker Red (60 nM) for 3 h. Baf A1 (100 nM, 24 h) and starvation (no serum, 24 h) were used as negative and positive control, respectively. **A** Representative images of LysoTracker Red (red) staining. Nuclei were visualized with Hoechst stain (blue). Scale bar 10 μm. **B** Quantification of LysoTracker Red staining intensities in A549, BEAS2B and 16HBE cells are displayed as the ratio of LysoTracker Red to Hoechst fluorescence intensity. Ten visual fields from each group were randomly selected to quantify the fluorescence intensity. **C** Calu-3, A549, BEAS2B and 16HBE cells were transfected with control empty vector or NSP6-encoding plasmid for 24 or 48 h. Whole-cell lysates were examined for cathepsin D (CathD) cleavage. **D**, **E** A549, BEAS2B and 16HBE cells were transfected with control empty vector or NSP6-encoding plasmid for 24 h then incubated with acridine orange (AO; 1 μM, 30 min). Baf A1 and starvation were used as negative and positive control, respectively. **D** Representative fluorescence images of AO staining. Scale bar 50 μm. **E** AO^+^ cells were quantified by flow cytometry. Results are displayed as percentages. The dashed lines indicate the gate between AO^-^ and AO^+^ cells. **F** Representative immunoblots of reciprocal immunoprecipitation (IP) of NSP6 and ATP6AP1. Calu-3, A549, BEAS2B and 16HBE cells were transfected with control empty vector or His-tagged NSP6-encoding plasmid for 20 h and then subject to IP by anti-His tag or anti-ATP6AP1 antibody or IgG (negative control). Seven percent of the lysate used for the IP was loaded as input (*n* = 3). **G** BEAS2B and 16HBE cells were transfected with control empty vector or NSP6-encoding plasmid for 44 h. Whole-cell lysates were examined for ATP6AP1 (Abcam; ab176609). Significance was assessed using one-way ANOVA with Tukey’s multiple comparison test. All the quantitative data are presented as means ± SD. **p < 0.01; ***p < 0.001; ns no significance.

We next investigated if NSP6 altered lysosome acidification by staining the cells with LysoTracker Red. Confocal microscopy revealed that both NSP6 and bafilomycin A1 markedly reduced the LysoTracker Red signals (Fig. 5A, B). The cleavage of pro-cathepsin D, which typically occurs under acidic conditions (pH 4–5) leading to the maturation of cathepsin D [21], was also used as an indicator of lysosome acidification. Consistently, prominent accumulation of pro-cathepsin D and decreased levels of mature cathepsin D upon NSP6 induction were observed (Fig. 5C). To confirm the disruption of lysosome acidification by NSP6, we further used a lysosomotropic metachromatic fluorochrome, acridine orange, which emits bright red fluorescence once protonated in acidic organelles (e.g. lysosomes) and green fluorescence in neutral environment (e.g. cytosol and nucleus) [22]. Concordantly, both confocal microscopy and flow cytometry analysis revealed that NSP6 reduced the proportions of cells bearing bright red fluorescence signals (Fig. 5D, E).

A recent proteomic study on the global interaction landscape of SARS-CoV-2 proteins uncovered a potential interaction between NSP6 and ATP6AP1 (an accessory subunit of vacuolar H^+^-ATPase for lysosomal acidification) [23]. Here, we validated NSP6-ATP6AP1 interaction by reciprocal co-immunoprecipitation in four human lung epithelial cell lines. Pull-down of His-tagged NSP6 with anti-His antibody or intact ATP6AP1 (~62–64 kDa) with a monoclonal antibody followed by detection of ATP6AP1 or NSP6, respectively, by Western blots confirmed the interaction between the two proteins (Fig. 5F). It is noteworthy that proteolytic processing of intact ATP6AP1 (~62–64 kDa) to its C-terminal (~42–44 kDa) and N-terminal (~20–22 kDa) fragments is required for its proper function [24]. Using another polyclonal antibody against ATP6AP1, we further observed that ATP6AP1 was almost entirely processed to its ~42–44 kDa form in control plasmid-transfected cells, whereas enforced expression of NSP6 impeded the cleavage of ATP6AP1 causing the accumulation of the intact form (~62–64 kDa) (Fig. 5G). These findings suggested that NSP6 might impair lysosome acidification by interacting with ATP6AP1 to block its cleavage-mediated activation.

### L37F NSP6 variant showed reduced abilities to activate inflammasomes and pyroptosis

**Fig. 6:**
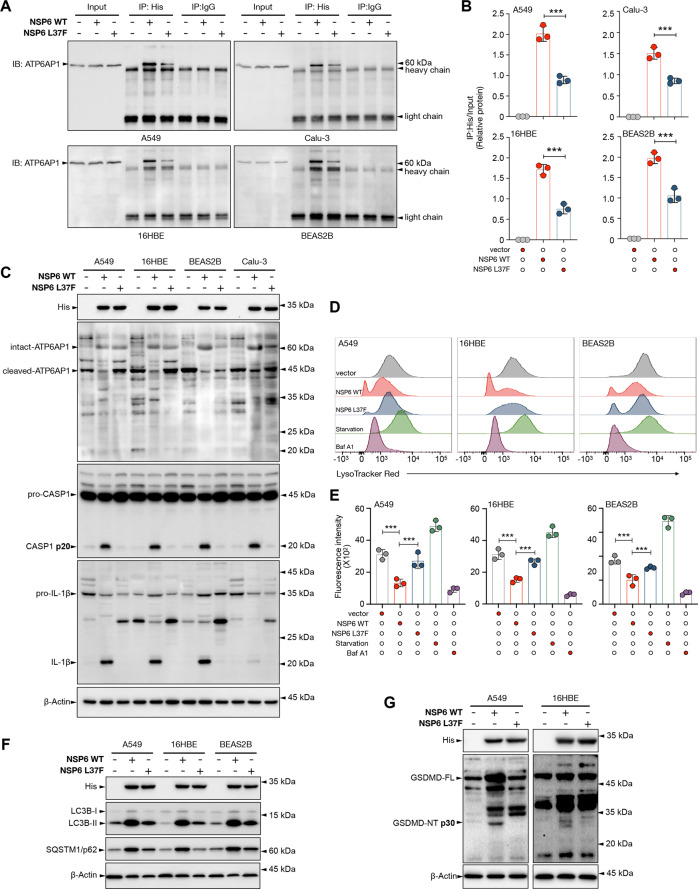
L37F NSP6 mutant failed to induce pyroptosis. **A–B** A549, Calu-3, 16HBE and BEAS2B cells were transfected with control empty vector or His-tagged wild-type (WT) or L37F NSP6-encoding plasmid for 24 h and then subject to immunoprecipitation (IP) by anti-His tag antibody or IgG (negative control). Five percent of the lysate used for the IP was loaded as input (*n* = 3). **A** Representative immunoblots of IP of WT or L37F NSP6 and ATP6AP1. **B** Quantification of relative protein levels is displayed as the ratio of intensity of IP:His to input. **C** A549, Calu-3, 16HBE and BEAS2B cells were transfected with control empty vector or His-tagged WT or L37F NSP6-encoding plasmid for 50 h. Whole-cell lysates were examined for His-tagged NSP6, inactive/full-length and active forms of ATP6AP1 (p45; Abcam; ab176609), CASP1 (p20), and IL-1β (p17). **D**–**F** A549, 16HBE and BEAS2B cells were transfected with control empty vector or His-tagged WT or L37F NSP6-encoding plasmid for 24 h. **D** Cells were then stained with LysoTracker Red and analyzed by flow cytometry. **E** Quantification of LysoTracker Red staining intensities (mean) in A549, 16HBE and BEAS2B cells. **F** Whole-cell lysates were examined for His-tagged WT or L37F NSP6, LC3B, SQSTM1/p62. **G** A549 and 16HBE cells were transfected with control empty vector or His-tagged WT or L37F NSP6-encoding plasmid for 48 h. Whole-cell lysates were examined for His-tagged NSP6, GSDMD (full-length and p30 NT fragment). **C**, **F–G** β-Actin was used as a loading control. Significance was assessed using one-way ANOVA with Tukey’s multiple comparison test. All the quantitative data are presented as means ± SD. ***p < 0.001.

A previous epidemiological analysis showed that the L37F NSP6 variant was associated with asymptomatic SARS-CoV-2 infection. To investigate if the lower pathogenicity of L37F NSP6 variant is linked to its altered ability to trigger pyroptosis, we overexpressed WT or L37F NSP6 in lung epithelial cells. Pull-down of His-tagged WT or L37F NSP6 with anti-His antibody showed that the L37F mutant had reduced interaction with the intact ATP6AP1 and was less efficacious in blocking the proteolytic processing of intact ATP6AP1 to its C-terminal functional form (Fig. 6A, B). The L37F mutant also failed to trigger the downstream inflammasome pathway as shown by the levels of CASP1 p20 and mature IL-1β (Fig. 6C). Moreover, we observed that L37F NSP6 had a significantly reduced ability to inhibit lysosome acidification as visualized by LysoTracker Red staining (Fig. 6D, E). Consistently, L37F NSP6-expressing cells showed a lesser extent of autophagic flux stagnation as shown by the reduced accumulation of LC3B-II and SQSTM1/p62 as compared to cells expressing WT NSP6 (Fig. 6F). Importantly, the L37F mutant failed to trigger pyroptosis (Fig. 6G).

### Pharmacological inhibition of caspase-1 or knockdown of NLRP3/ASC/Caspase-1 abolished NSP6-induced pyroptosis

**Fig. 7:**
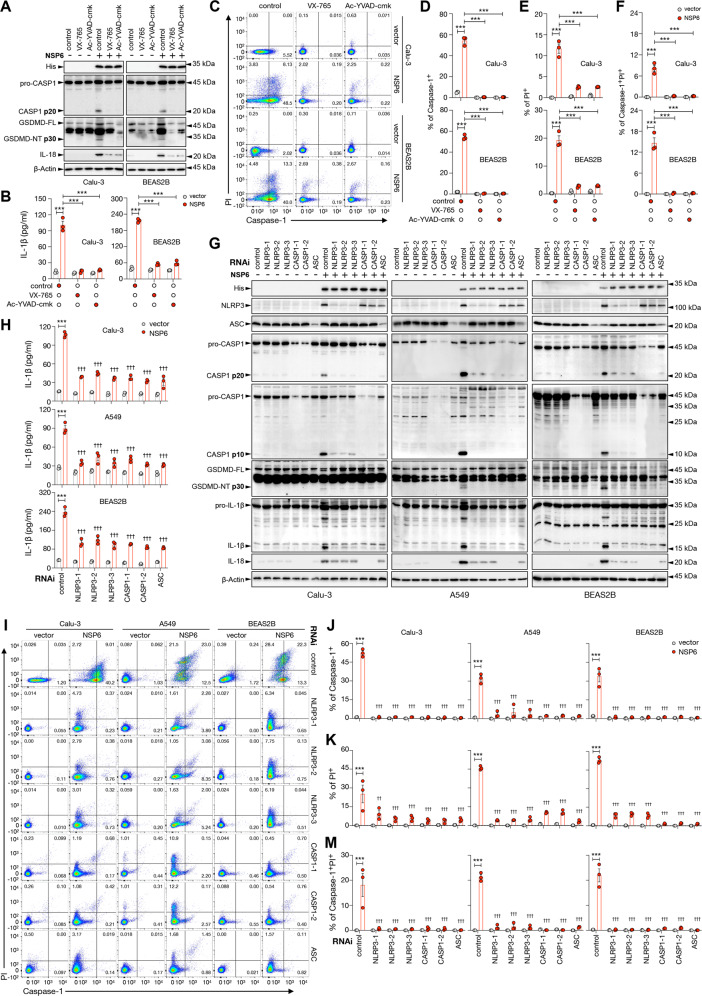
NLRP3, ASC and CASP1 were required for NSP6-induced pyroptosis. **A–F** Calu-3 and BEAS2B cells were pretreated without or with VX-765 (10 μM) or Ac-YVAD-cmk (100 μM) for 3 h and then transfected with control empty vector or NSP6-encoding plasmid in the same culture for another 24 h. **A** Whole-cell lysates were examined for His-tagged NSP6, inactive/full-length and active forms of CASP1 (p20), and GSDMD (p30 NT fragment). **B** Quantification of IL-1β in culture supernatants by ELISA showed that VX-765 or Ac-YVAD-cmk treatment attenuated NSP6-induced IL-1β secretion. **C** Cell death monitored by propidium iodide (PI) staining and active CASP1 identified by FLICA probe were analyzed by flow cytometry. Quantification data of CASP1^+^ (**D**), PI^+^ (**E**) and CASP1^+^PI^+^ (**F**) cells are displayed as percentages. (**G–M**) Calu-3, A549 and BEAS2B cells were transfected with scrambled control siRNA or three different NLRP3-specific siRNAs, two different CASP1-specific siRNAs and one ASC-specific siRNA for 24 h followed by transfection with control empty vector or NSP6-encoding plasmid for another 24 h. **G** Whole-cell lysates were examined for His-tagged NSP6, NLRP3, ASC, IL-18, inactive/full-length and active forms of CASP1 (p20), GSDMD (p30 NT fragment) and IL-1β (p17). **A**, **G** β-Actin was used as a loading control. **H** Quantification of IL-1β in culture supernatants showed that NLPR3, CASP1, ASC knockdown attenuated NSP6-induced IL-1β secretion. **I** Cell death monitored by PI staining and active CASP1 identified by FLICA probe were analyzed by flow cytometry. Quantification data of CASP1^+^ (**J**) PI^+^ (**K**) and CASP1^+^PI^+^ (**M**) cells are displayed as percentages. Significance was assessed using two-way ANOVA with Sidak’s multiple comparison test. All the quantitative data are presented as means ± SD. ***p < 0.001; ^†^*p* < 0.05^; ††^*p* < 0.01; ^†††^*p* < 0.001 when compared to untreated or control siRNA-transfected NSP6 group.

Next, we employed two pharmacological inhibitors of caspase-1, namely VX-765 and Ac-YVAD-cmk [25], to determine if caspase-1 is required for NSP6-induced cell death. Both agents efficiently prevented the generation of p20 subunit of caspase-1 and GSDMD-NT, and IL-18 maturation (Fig. 7A) as well as the release of IL-1β into the supernatants (Fig. 7B). Flow cytometry confirmed the complete abolition of caspase-1 activity and pyroptosis by both inhibitors. Nevertheless, the reduction of propidium iodide-positive cells by caspase-1 inhibitors was not complete, indicating the involvement of other forms of cell death in the action of NSP6 (Fig. 7C–F). Similarly, siRNAs targeting the inflammasome components NLRP3, ASC or caspase-1 phenocopied the effects of caspase-1 inhibitors on NSP6-induced maturation of gasdermin D and IL-18 (Fig. 7G), IL-1β release (Fig. 7H), and pyroptosis (Fig. 7I–M). Knockdown of GSDMD also attenuated NSP6-induced cell death as shown by the reduction propidium iodide-positive cells (Supplementary Fig. S3). To determine if the canonical apoptotic pathway could be involved in NSP6-induced cell death, we measured total and cleaved caspase-3. However, NSP6 did not significantly alter caspase-3 cleavage (Supplementary Fig. S4).

### 1α,25-Dihydroxyvitamin D_3_, metformin and polydatin attenuated NSP6-induced autophagic flux impairment, inflammasome activation and pyroptosis

We and other investigators have shown that 1α,25-dihydroxyvitamin D_3_ (active form of vitamin D_3_), metformin (an anti-diabetic drug) or polydatin (a phytochemical) could alleviate autophagic flux impairment in disease context through different mechanisms [26–28]. We therefore explored if these pharmacological agents could protect against NSP6-induced inflammasome activation and pyroptosis. The restoration of autophagic flux was confirmed by the reduced accumulation of non-digestive autophagosomes in the mCherry-GFP-LC3B assay (Supplementary Fig. S5A, B). The restoration of lysosome acidification by 1α,25-dihydroxyvitamin D_3_, similar to our previous report [26], was also verified by acridine orange staining (Supplementary Fig. S5C, D). Importantly, these agents prevented NSP6-induced caspase-1 cleavage, GSDMD-NT formation, IL-1β/IL-18 maturation (Supplementary Fig. S5E), IL-1β release (Supplementary Fig. S5F), and pyroptosis (Supplementary Fig. S5G, H). In contrast, chloroquine (a lysosome inhibitor) exerted opposite effects (Supplementary Fig. S5E–H). It is noteworthy that, as compared to the effect of caspase-1 inhibition, all three autophagic reactivators failed to completely abrogate NSP6-induced non-pyroptotic cell death (Supplementary Fig. S5G, H). Consistent with the protective role of autophagy against ROS, these agents also attenuated NSP6-induced ROS production (Supplementary Fig. S6).

### SARS-CoV-2 triggered NLRP3 inflammasome activation and pyroptosis and their reversal by NSP6 siRNA and 1α,25-dihydroxyvitamin D_3_

To verify our findings of NSP6-induced autophagic flux impairment and the downstream NLRP3 inflammasome-dependent pyroptosis in human airway epithelial cells, we transfected A549 cells with three different siRNA duplexes targeting the NSP6 sequence or scrambled control siRNA prior to infection with live SARS-CoV-2. SARS-CoV-2 significantly induced the accumulation of LC3B-II and p62, whereas knockdown of NSP6 abolished the accumulation. Meanwhile, SARS-CoV-2 strongly induced the expression of NLRP3 and the activation of CASP1 and GSDMD, all of which could be attenuated by NSP6 knockdown (Supplementary Fig. S7A). In line with our observation that 1α,25-dihydroxyvitamin D_3_ suppressed NSP6-mediated activation of inflammasome and pyroptosis, this chemical protected A549 cells from SARS-CoV-2-induced CASP1 and GSDMD activation (Supplementary Fig. S7B).

## Discussion

More than a year after its emergence, SARS-CoV-2 continues to plague the world and dominate our daily lives. Even with the development of vaccines, SARS-CoV-2 variants bring concerns for the increased spread and the escape from both vaccine and natural immunity [29]. Given that NSP6 mutation developed as the virus spread and is linked to viral pathogenicity [11, 30], it is crucial to understand the role of NSP6 in COVID-19 pathogenesis.

In this study, we found that NLRP3 inflammasome activation caused by autophagic flux stagnation manifested as concomitant accumulation of LC3B-II and SQSTM1/p62 plays a key role in NSP6-induced pyroptosis in lung epithelial cells. Nevertheless, on top of the direct inhibition on lysosome acidification, it is unclear at this point if NSP6 could induce autophagosome formation via the canonical autophagy components (e.g. ULK1, VPS34) or the non-canonical v-ATPase-ATG16L1 pathway to convert LC3-I to LC3-II [31]. Another interesting observation is that neither pharmacological inhibition of caspase-1 nor silencing of NLRP3, ASC or caspase-1 completely abolished NSP6-induced cell death, suggesting other cell death mechanisms are also at work. To this end, inhibition of autophagy via conditional deletion of *Becn1* has been shown to activate the canonical apoptotic signaling during lung development [32]. Our recent study also showed that impairing lysosomal function enhanced chemotherapeutic-induced apoptosis in lung cancer cells [33]. It is therefore likely that apoptosis might mediate part of the cytotoxic effect of NSP6. On the other hand, lysosome dysfunction and the resulting autophagic flux impairment have been linked to activation of other downstream pro-inflammatory signaling cascades, such as AIM2 inflammasomes [34], SQSTM1/p62-nuclear factor κB signaling [35] and cyclic GMP AMP synthase (cGAS)-STING pathway [36]. It would be equally important to investigate the potential involvement of these pro-inflammatory pathways in the pathogenicity of NSP6.

Lysosomes are the primary degradative organelles in eukaryotic cells, which are essential for the efficient removal of protein aggregates, damaged organelles, and internalized pathogens. Its digestive function is executed by hydrolytic enzymes that are activated in an acidic medium (pH between 4.5–5.5) [37]. Therefore, normal lysosomal acidification is extremely important for its degradative function. Consistent with previous works showing that SARS-CoV and MERS-CoV could neutralize endosomal/lysosomal pH [6, 38, 39], our present study has uncovered the inhibitory effect of SARS-CoV-2 NSP6 on lysosomal acidification via interacting with ATP6AP1, thereby causing autophagic flux stagnation. Accordingly, impairment of the lysosomal acidic environment by chloroquine resulted in enhanced NSP6-induced caspses-1 cleavage, GSDMD-NT formation, IL-1β/IL-18 maturation, IL-1β release, and pyroptosis. Our present findings may in part explain the results of a meta-analysis of randomized trials showing an increased mortality among COVID-19 patients treated with hydroxychloroquine, a chloroquine derivative that also inhibits the lysosome [40]. It would be important to examine if other NSPs of SARS-CoV-2 could activate the same pathway to mediate the pathogenesis of COVID-19. Moreover, it is worth noting that, in another study [12], overexpression of NSP6 did not induce autophagy. However, that experiment was performed in human embryonic kidney HEK293T cells but not in airway epithelial cells. The use of airway epithelial cells as the disease model should be more relevant to the pathogenesis of COVID-19.

With relevance to potential treatments, we demonstrated that 1α,25-dihydroxyvitamin D_3_, metformin and polydatin could restore the autophagic flux to protect against NLRP3 inflammasome activation and pyroptosis in NSP6-overexpresssing cells. Indeed, these agents have been shown to restore autophagic flux in other pathological conditions. For instance, our previous study showed that 1α,25-dihydroxyvitamin D_3_ could reactivate the autolysosomal degradation functions of gastric epithelial cells to protect against intracellular *Helicobacter pylori* infection through the PDIA3 receptor [26]. We also reported that polydatin could ameliorate non-alcoholic steatohepatitis by restoring the lysosomal acidification in hepatocytes by upregulating TFEB [28]. Other investigators also demonstrated that metformin could restore autophagic flux in melanoma cells and saturated fatty acid-challenged endothelial cells via a TRIB3- and AMPK-dependent mechanism, respectively [27, 41]. Although it is thought that the molecular signaling of 1α,25-dihydroxyvitamin D_3_, metformin and polydatin are different, our present study indicates that their intracellular signaling could influence the NLRP3 inflammasomes via restoring the autophagic flux. Further clinical studies are therefore warranted to confirm the therapeutic effects of these agents. It is noteworthy that, although a recent clinical trial showed that a single high dose of vitamin D_3_ did not alter the length of hospital stay among patients with COVID-19 [42], the hepatic expression of CYP2R1 and CYP27A1, enzymes that convert vitamin D_3_ to 25-hydroxyvitamin D_3_ may be downregulated during critical illness, and therefore administration of vitamin D_3_ might fail to increase the level of 25-hydroxyvitamin D_3_ or 1α,25-dihydroxyvitamin D_3_ [43]. Thus the therapeutic effect of the bioactive form of vitamin D_3_ (i.e., 1α,25-dihydroxyvitamin D_3_) instead of its precursors should be examined.

In summary, our results demonstrate that NSP6 of SARS-CoV-2 can impair lysosome acidification in lung epithelial cells via interacting with the vacuolar type H^+^-ATPase component ATP6AP1, thereby causing autophagic flux stagnation to instigate NLRP3 inflammasome activation and pyroptosis. Pharmacological restoration of autophagic flux by 1α,25-dihydroxyvitamin D_3_, metformin or polydatin might represent a novel therapeutic strategy to mitigate the hyperinflammatory state associated with COVID-19 and improve clinical outcomes.

## Materials and methods

### Chemicals, reagents and antibodies

VX-765 was purchased from InvivoGen (San Diego, CA, USA). Ac-YVAD-cmk (SML0429), adenosine 5′-triphosphate (ATP; A2383), bafilomycin A1 (B1793), chloroquine (C6628), 1α,25 dihydroxyvitamin D_3_ (1,25D_3_, D1530), metformin (1396309) and polydatin (15721) were purchased from Sigma-Aldrich (St. Louis, MO, USA). Dynabeads Protein G (10004D), LysoTracker Deep Red (12492), acridine orange (A3568), Lipofectamine 3000 Reagent (L3000015), Lipofectamine RNAiMAX Transfection Reagent (13778075) and human interleukin-1beta (IL-1β) ELISA kit (88-7261-88) were purchased from Thermo Fisher Scientific (Waltham, MA, USA). FAM-FLICA-Caspase-1 Assay Kit was obtained from Immunochemistry (Bloomington, MN, USA). Human Cytokeratin 18 (M65) ELISA Kit (ab227896) was obtained from Abcam (Cambridge, MA, USA). Primary antibodies – Anti-6x-His tag (R930-25) was purchased from Thermo Fisher Scientific; anti-6x-His tag (2365), anti-caspase-1 p20 (2225), anti-caspase-1 (3866), anti-IL-18 (54943), anti-Cathepsin D (69854), anti-LAMP1 (9091), anti-NLRP3 (13158), anti-ASC (13833), anti-Gasdermin D (96458), and anti-LC3B (3868) were purchased from Cell Signaling Technology (Danvers, MA, USA); anti-caspase-1 p10 (sc-514), anti-caspase-1 (sc-56036), anti-IL-1β (sc-7884), anti-ATP6AP1 (sc-81886), anti-caspase-3 (sc-56052) and anti-β-Actin (sc-47778) were purchased from Santa Cruz Biotechnology (Dallas, Texas, USA); anti-SQSTM1/p62 (610832) was purchased from BD Biosciences (New Jersey, USA); anti-ATP6AP1 (ab176609) was purchased from Abcam. Secondary antibodies: anti-mouse conjugated to horseradish peroxidase (7076) and anti-rabbit conjugated to horseradish peroxidase (7074) for immunoblotting were purchased from Cell Signaling Technology. Anti-rabbit conjugated to Alexa Fluor 568 (A11011) and anti-mouse conjugated to Alexa Fluor 647 (A32728) for confocal microscopy were purchased from Sigma-Aldrich.

### Cell lines and medium

Human bronchial and lung epithelial cell lines were purchased from ATCC. 16HBE was a kind gift from Dr. Wing Hung Ko (The Chinese University of Hong Kong, Hong Kong). BEAS2B were cultured in complete Keratinocyte Serum Free medium (K-SFM, Gibco, Carlsbad, CA, USA) formulated with 0.05 mg/ml BPE, 0.1 ng/ml EGF, 0.4 mM calcium chloride and 1% penicillin/streptomycin. 16HBE, A549 and Calu-3 were cultured in Minimum Essential Medium (MEM), Ham’s F-12 Nutrient Mixture and Dulbecco’s Modified Eagle Medium: Nutrient Mixture F-12 (DMEM/F-12) medium (Gibco) supplemented with 10% FBS (Gibco) and 1% penicillin/streptomycin, respectively. All experiments were carried out on BEAS2B between passage 2 and 20, 16HBE between passage 7 and 25, A549 between passage 20 and 30, Calu-3 between passage 10 and 30. All cell models were regularly tested for mycoplasma contamination.

### Clinical specimens

Serum samples from 157 COVID-19 patients and 52 healthy subjects were obtained from Shenzhen Center for Disease Control and Prevention and stored at −80 °C. Serum samples were heat-inactivated at 56 °C for 30 min prior to analysis. The detailed demographics and baseline characteristics of these patients was shown in Supplementary Table S1. All patients were admitted to the Third People’s Hospital of Shenzhen. The study was approved by the institutional Ethics Committee. The requirement for informed consent was waived as described previously [44].

### Determining COVID-19 disease severity

A severity scale for COVID-19 was stratified by the primary outcome of any supplemental oxygen usage during the hospitalization to maintain resting oxyhemoglobin saturation ≥90%. The defined categories are as follows: (1) mild disease, based on normal oxyhemoglobin saturation but with symptoms of pneumonia; (2) moderate disease: based on use of supplemental oxygen at ≤8 L/min; (3) severe disease: based on requirement of high-flow nasal cannula oxygen therapy, noninvasive or invasive ventilation or extracorporeal membrane oxygenation.

### SARS-CoV-2 (live virus) infection

SARS-CoV-2 (Guangdong/20SF014/2020|EPI_ISL_403934) was used to infect A549 cells as previously described [45]. In brief, virus stock was diluted in DMEM medium supplied with 2% FBS. Titer was determined by plaque titration assay. The virus was inoculated into A549 cells at a multiplicity of infection (MOI) of 1 for 48 h. Infection medium was removed 1 h post adsorption and cells were washed 2 times in PBS to remove unattached virus, then cultured in fresh DMEM medium supplied with 10% FBS until 48 h. Mock control was prepared by inoculating cells with DMEM supplied with 2% FBS, in accordance to virus stock preparation. All infection experiments were carried out under biosafety level 3 conditions with enhanced respiratory personal protection equipment.

### Plasmid construction and transfection

The sequences of NSP1, NSP2 and NSP6 of SARS-CoV-2 (Wuhan-Hu-1 isolate, GenBank: MN908947.3) were synthesized and cloned into pcDNA3.1(+)-C-6His vector using BamHI and ApaI cloning sites by GenScript. The translational protein sequence of NSP1: MESLVPGFNEKTHVQLSLPVLQVRDVLVRGFGDSVEEVLSEARQHLKDGTCGLVEVEKGVLPQLEQPYVFIKRSDARTAPHGHVMVELVAELEGIQYGRSGETLGVLVPHVGEIPVAYRKVLLRKNGNKGAGGHSYGADLKSFDLGDELGTDPYEDFQENWNTKHSSGVTRELMRELNGG.

The translational protein sequence of NSP2: AYTRYVDNNFCGPDGYPLECIKDLLARAGKASCTLSEQLDFIDTKRGVYCCREHEHEIAWYTERSEKSYELQTPFEIKLAKKFDTFNGECPNFVFPLNSIIKTIQPRVEKKKLDGFMGRIRSVYPVASPNECNQMCLSTLMKCDHCGETSWQTGDFVKATCEFCGTENLTKEGATTCGYLPQNAVVKIYCPACHNSEVGPEHSLAEYHNESGLKTILRKGGRTIAFGGCVFSYVGCHNKCAYWVPRASANIGCNHTGVVGEGSEGLNDNLLEILQKEKVNINIVGDFKLNEEIAIILASFSASTSAFVETVKGLDYKAFKQIVESCGNFKVTKGKAKKGAWNIGEQKSILSPLYAFASEAARVVRSIFSRTLETAQNSVRVLQKAAITILDGISQYSLRLIDAMMFTSDLATNNLVVMAYITGGVVQLTSQWLTNIFGTVYEKLKPVLDWLEEKFKEGVEFLRDGWEIVKFISTCACEIVGGQIVTCAKEIKESVQTFFKLVNKFLALCADSIIIGGAKLKALNLGETFVTHSKGLYRKCVKSREETGLLMPLKAPKEIIFLEGETLPTEVLTEEVVLKTGDLQPLEQPTSEAVEAPLVGTPVCINGLMLLEIKDTEKYCALAPNMMVTNNTFTLKGG.

The translational protein sequence of NSP6: SAVKRTIKGTHHWLLLTILTSLLVLVQSTQWSLFFFLYENAFLPFAMGIIAMSAFAMMFVKHKHAFLCLFLLPSLATVAYFNMVYMPASWVMRIMTWLDMVDTSLSGFKLKDCVMYASAVVLLILMTARTVYDDGARRVWTLMNVLTLVYKVYYGNALDQAISMWALIISVTSNYSGVVTTVMFLARGIVFMCVEYCPIFFITGNTLQCIMLVYCFLGYFCTCYFGLFCLLNRYFRLTLGVYDYLVSTQEFRYMNSQGLLPPKNSIDAFKLNIKLLGVGGKPCIKVATVQ. NSP6 L37F point mutation was generated using QuikChange II XL Site-Directed Mutagenesis Kit (Agilent) and verified by sequencing. All plasmid transfections were performed using Lipofectamine 3000 Reagent (Thermo Fisher Scientific) according to the manufacturer’s protocol in Opti-MEM medium. Transfection medium of 16HBE, A549 and Calu-3 cells were replaced by RPMI 1640 medium (Gibco) supplemented with 10% FBS and 1% L-Glutamine 6 h post transfection. Transfection medium of BEAS2B cells was replaced by K-SFM supplemented with 0.05 mg/ml BPE, 0.1 ng/ml EGF, 0.4 mM calcium chloride and 1% L-glutamine 6 h post transfection. Cells were then incubated at 37 °C in CO_2_ incubator until further examination.

### RNA interference (RNAi)

Lipofectamine RNAiMAX Transfection Reagent was used to deliver the indicated siRNA duplexes into cultured cells and knockdown experiment was performed following the manufacturer’s protocol for 24 h. Then the indicated siRNA duplexes were dually co-transfected with plasmids into cells using Lipofectamine 3000 Reagent for another 24 h. Transfection medium was then replaced by RPMI 1640 or K-SFM medium 6 h post transfection. siRNA duplexes were synthesized by GenePharma and the sequences are as follows: CASP1-1: GGCAGAGAUUUAUCCAAUATT, ASC: GCUUCUACCUGGAGACCUATT, NLRP3-1: AGGUGUUGGAAUUAGACAATT [46], NLRP3-2: AAGCTTCAGGTGTTGGAATTA, NLRP3-3: GCATGATCTCTCAGCAAAT [47], CASP1-2: GACUCAUUGAACAUAUGCA, control: UUGUACUACACAAAAGUACUGTT. There different siRNAs against SARS-CoV-2 NSP6 and the scrambled control siRNA were designed and provided by RiboBio (Guangzhou, China).

### Western blots

In general, cells were harvested in lysis buffer (25 mM Tris-HCl, pH 7.5; 150 mM NaCl; 5 mM EDTA; 1% Triton X-100; protease inhibitor cocktail on ice for 15 min and resolved in 3x sample buffer (187.5 mM Tris-HCl, pH 6.8; 6% (w/v) SDS; 30% glycerol; 0.03% (w/v) bromophenol blue; 125 mM DTT) to 1x final concentration and incubated at 95 °C for 5 min, then analyzed on 10%, 12% or 15% SDS-polyacrylamide gels and transferred to 0.2-μm nitrocellulose membranes. Membrane was blocked then incubated with primary antibodies in blocking buffer (PBS, pH 7.4 or TBS pH 7.6; 5% BSA; 0.05% Tween-20) at 4 °C with shaking overnight. Antibodies were detected using horseradish peroxidase (HRP)-conjugated secondary antibody for 1 h at room temperature. 0.1% TBST was used as washing buffer. Appropriate bands were detected using WesternBright ECL HRP substrate from Advansta (K-12045-D50, San Jose, CA, USA). The BioRad ChemDoc Imaging System was used for visualization of bands.

### Co-Immunoprecipitation

Dynabeads Protein G was used to capture antibody-protein complex. Cells were seeded onto 10 cm culture dish at appropriate density with 80% confluence 12 h prior to transfection. Plasmids were transfected as described above for 20 h. Cells were lysed in 1 ml lysis buffer with 1:50 protease inhibitor cocktail on ice for 15 min. Lysate was centrifuged at 12,000 rpm, 4 °C for 15 min. Supernatant was collected and divided into three tubes. One tube containing 1/15 volume was used as input and resolved in 3× sample buffer (to 1×) then denatured at 95 °C for 5 min. The other two tubes containing the same amount of supernatants were used for immunoprecipitation with one adding 0.5 μg of the indicated antibody and the other one adding 0.5 μg of IgG control. The supernatant of cell lysate with antibody or IgG was rotated at 4 °C overnight then incubated with 10 μl of Dynabeads and rotated at 4 °C for 1 h. The beads-antibody-protein complex was collected by magnetic rack and washed 5 times using ice-cold lysis buffer with gentle shaking. The beads pellet was resolved in 2x sample buffer and denatured at 95 °C for 5 min then analyzed by Western blots.

### FLICA-caspase-1 and propidium iodide staining

FAM-YVAD-FMK probe, propidium iodide, Hoechst stain, 10× apoptosis washing buffer and fixative reagents are supplied in the FAM-FLICA-Caspase-1 Assay Kit. Staining was performed following the manufacturer’s instruction. In brief, cells were subcultured in 12-well plates before transfection or exposure to the indicated experimental conditions. 30× FLICA solution at a ratio of 1:30 was added to cells at 37 °C for 3 h incubation under the experimental conditions and washed with 1× apoptosis washing buffer. Cells were then stained with propidium iodide for 5 min at room temperature following 2 times washing and resuspended in 300 μl of 1× apoptosis washing buffer and immediately analyzed by flow cytometry on BD Aria Fusion Cell sorter and Cell Analyzer. The percentage of caspase-1-positive and propidium iodide-positive cells were determined by FlowJo software. For microscopy, cells were stained with FLICA probe as described above, then dually stained with 0.5% v/v hoechst for 5 min at room temperature and washed with 1× apoptosis washing buffer then fixed with kit-supplied fixative. Fixed cells were mounted with 80% glycerol and imaged under Olympus FV1200 confocal laser scanning microscope. For propidium iodide uptake assay, cells were stained with propidium iodide for 5 min at room temperature following 3 times washing in PBS and fixed with 100% ice-cold methanol at −20 °C for 10 min then dually stained with 0.5% (v/v) Hoechst for 5 min at room temperature. Fixed cells were mounted with 80% glycerol and imaged under Leica TCS SP8 Inverted Confocal Microscope.

### LysoTracker red staining

Cells were seeded onto glass coverslips in 12-well plate prior to transfection or treatment as indicated then stained with LysoTracker Red (60 nM) under experimental conditions at 37 °C for 3 h, followed by staining with 0.5% v/v hoechst for 5 min at room temperature. Cells were washed 3 times in PBS and fixed with 100% ice-cold methanol at −20 °C for 10 min then washed 3–5 times in PBS. Fixed cells were mounted with 80% glycerol and imaged under Leica TCS SP8 Inverted Confocal Microscope. For flow cytometry analysis, cells were stained with LysoTracker Red and then trypsinized with 0.05% Trypsin-EDTA and resuspended in 1× PBS then immediately analyzed on BD Aria Fusion Cell sorter and Cell Analyzer in PE-Cy5-A channel. Fluorescence intensity (mean) was quantified using FlowJo software.

### Acridine orange staining

Cells were transfected and/or treated as indicated then stained with Acridine Orange (1 ug/ml) under experimental conditions at 37 °C for 30 min then washed once in 1× PBS. Cells were trypsinized with 0.05% Trypsin-EDTA and resuspended in 1× PBS then immediately analyzed by flow cytometry. The red fluorescence was measured in PercP/Cy5 channel on BD Aria Fusion Cell sorter and Cell Analyzer. The percentages of Acridine Orange positive (AO^+^) cells were quantified using FlowJo software. For microscopy, cells were seeded onto glass coverslips in 12‐well plate prior to transfection and/or treatment then stained with Acridine Orange as described above and evaluated immediately under Leica DMI6000 Inverted microscope.

### Immunofluorescence

Cells were seeded onto glass coverslips in 12-well plate prior to transfection and/or treatment as indicated then washed once in 1× PBS and fixed with 100% ice-cold methanol at −20 °C for 10 min, followed by 3–5 times washing in 1× PBS. Cells were blocked and incubated with antibody (His-tag, 1:150; LAMP1, 1:200) in blocking buffer (0.1 % BSA and 5% goat serum in PBS, pH 7.4) at 4 °C overnight. Then cells were washed and incubation with Alexa Fluor 647 or 568 antibody (1:300) for 1 h then DAPI for 5 min at room temperature avoiding light, followed by 3–5 times washing in 1× PBS. Cells were mounted with 80% glycerol and imaged under Leica TCS SP8 Inverted Confocal Microscope. The colocalization coefficient was measured using the ‘Colocalization/coloc 2’ plugin of ImageJ-Fiji.

### Scanning electron microscopy

Cells were seeded onto 10 mm glass coverslips in 24-well plate prior to transfection as indicated and fixed with 2.5% glutaraldehyde in PBS for 1 h. Fixed cells were washed 3 times in PBS then post-fixed in 1% OsO_4_ for 1 h following 3 times washing in ddH_2_O and dehydrated through ethanol series (70%, 80%, 95%, 100%). The dehydrated cells were critical-point dried and mounted on stubs then sputter coated with a thin layer of conductive metal (gold-palladium) then viewed under the Hitachi SU8010 scanning electron microscope.

### Cytokine and M65 measurement

Human IL-1β enzyme-linked immunosorbent assay (ELISA) kit was used to detect IL-1β levels in cell culture supernatants according to the manufacturer’s instruction. In brief, the ELISA plate was coated with the capture antibody in coating buffer and incubated one night before measurement at 4 °C. After washing and blocking, 100 μl of 1:3 or 1:5 diluted samples were added to appropriate wells and incubated overnight at 4 °C. After incubation with secondary antibody and HRP, OD value at 570 nm was subtracted from OD value at 450 nm. Measurement of circulating IL-1β and IL-18 levels in serum of COVID-19 patients were previously described [14]. Human Cytokeratin 18 (M65) ELISA Kit was used to detect human circulating M65 levels in COVID-19 patients and healthy subjects according to the manufacturer’s protocol.

### Transcriptome analysis

RNA sequencing datasets of SARS-CoV-2 infection in human normal bronchial and malignant cell lines (A549, Calu-3, NHBE) were downloaded from the Gene Expression Omnibus (GEO) within the series GSE147507 [48]. Bulk RNA-Seq data of lung tissue of COVID-19 patients or uninfected individuals were downloaded within the series GSE171668 [49] and GSE175779, respectively. RNA-Seq raw reads of patients infected with SARS-CoV-2 for quantitating NSP6 expression level were derived from the series GSE163959 [50]. RNA sequencing data were first evaluated by FastQC with the default parameter and then aligned with Hisat2 against the human (GRCh38) genome and SARS-CoV-2 genome (NC_045512) guided by GENCODE gene annotation and NCBI RefSeq SARS-CoV-2 genome annotation with the default parameter. The abundance of genes in each sample was calculated by featureCounts packages [51] with the “-M” parameter. DEGs were estimated by using R package DESeq2 [52] with the following condition: adjusted *p*-value < 0.05 and the absolute value of log_2_ fold-change >1.

### Pathway enrichment analysis

Enrichment analysis of GO were conducted using R package clusterProfiler [53] and KEGG pathway annotation was performed on the list of DEGs by using KOBAS online analysis database to confirm if the genes are enriched in specific terms. The Benjamini and Hochberg method are used to correct the *P* values for the false discovery rate (FDR). GSEA was performed on all genes to identify the possible pathway of the SARS-CoV-2 infection by using R package clusterProfiler gseKEGG. Gene sets with adjusted *p* value < 0.05 were considered to be significantly enriched. Genes were ranked based on the product of the log_2_ fold change and the log_10_ moderated *t*-test *p* value between the SARS-CoV-2 and mock groups.

### Quantitation and statistical analysis

Statistical analysis was performed using the Prism 8 software (GraphPad, San Diego, CA, USA). For all statistical analysis a *p* value of <0.05 was determined to be significant. All data from cell models is presented as mean ± SD of at least three biological replicates. Patient characteristics were summarized using the standard descriptive statistics: median (IQR) for univariate and multivariate Cox regression analysis. M65 levels was log_2_ transformed to render the parametric statistical analyses. A cutoff point (90.7 pg/ml) for M65 was determined by maximized Youden Index Score. *p* values are depicted in all figures. For the comparison of two groups, the Student’s *t*-test or Wilcoxon log-rank was used. For the comparison of more than two groups, one-way ANOVA followed by Turkey’s multiple comparison test or two-way ANOVA with Sidak’s multiple comparison test or Kruskal-Wallis with Wilcox log-rank test was used. For the correlation coefficient between serum log_2_ M65 and IL-1β, serum levels of IL-1β and IL-18, Spearmen correlation test with 95% confidence interval was used.

## Supplementary information

Supplementary Table S1

Supplementary Figure Legends

Supplementary Figure S1

Supplementary Figure S2

Supplementary Figure S3

Supplementary Figure S4

Supplementary Figure S5

Supplementary Figure S6

Supplementary Figure S7

Reproducibility Checklist

Change of authorship request form

## Data Availability

The data that support the findings of this study are available from the corresponding author, WKKW, upon reasonable request.
