# Supplementary material for: SARS-CoV-2 non-structural protein 6 triggers NLRP3-dependent pyroptosis by targeting ATP6AP1
Source: Cell Death Differ. 2022 Jan 8;29(6):1240–54. doi: 10.1038/s41418-021-00916-7 (PMC9177730; doi:10.1038/s41418-021-00916-7)
Supplement: Supplementary file 1 — Supplementary Table S1 [file 41418_2021_916_MOESM1_ESM.docx]

| Table S1. Demographic and epidemiologic characteristics within 48 h of admission for the patients included in Kaplan-Meier curve | | | | |
| --- | --- | --- | --- | --- |
|  | Never used O_2_ | Required O_2_ | OR (95%CI) | p-value |
|  | N = 23 | N = 16 | Univariate | |
| **Patient characteristics** |  |  |  |  |
| Male sex – no. (%) | 11 (47.8) | 8 (50.0) | 1.1 (0.3-3.9) | 0.894 |
| Median age (IQR), years | 39.00 [28.50,49.50] | 48.50 [36.50,57.75] | 1.0 (0.99-1.1) | 0.081 |
| Age > 50 years | 5 (21.7) | 7 (43.8) | 2.8 (0.7-11.3) | 0.149 |
| Median heart rate (IQR) – beats/min | 89.00 [80.00,92.50] | 86.00 [78.75,93.75] | 1.0 (0.9-1.1) | 0.835 |
| Median respiratory rate (IQR) – breaths/min | 20.00 [19.00,20.00] | 20.00 [19.00,20.25] | 1.4 (0.8-2.3) | 0.229 |
| Median systolic blood pressure (IQR) – mmHg | 119.00 [111.50,131.00] | 126.00 [118.50,139.00] | 1.0 (0.99-1.1) | 0.193 |
| Median diastolic Blood Pressure (IQR) – mmHg | 80.00 [72.00,87.50] | 79.00 [74.00,83.75] | 1.0 (0.99-1.1) | 0.688 |
| Median temperature (IQR) – °C | 36.80 [36.60,37.15] | 36.80 [36.80,37.12] | 0.9 (0.2-3.5) | 0.850 |
| **Epidemiology – no. (%)** |  |  |  |  |
| Living in Hubei | 6 (26.1) | 1 (6.2) | 0.2 (0-1.8) | 0.143 |
| Recently visited to Hubei | 6 (26.1) | 5 (31.2) | 1.3 (0.3-5.3) | 0.725 |
| Had contact with Hubei residents | 4 (17.4) | 2 (12.5) | 0.7 (0.1-4.2) | 0.678 |
| **Principle symptoms at admission – no. (%)** |  |  |  |  |
| Fever | 17 (73.9) | 12 (75.0) | 1.1 (0.2-4.6) | 0.939 |
| Cough | 8 (34.8) | 5 (31.2) | 0.9 (0.2-3.3) | 0.818 |
| Sputum | 4 (17.4) | 2 (12.5) | 0.7 (0.1-4.2) | 0.678 |
| Nasal congestion | 0 (0.0) | 2 (12.5) | --- | 0.992 |
| Rhinorrhea | 2 (8.7) | 1 (6.2) | 0.7 (0.1-8.4) | 0.779 |
| Sore throat | 4 (17.4) | 3 (18.8) | 1.1 (0.2-5.7) | 0.913 |
| Chest pain or tightness | 1 (4.3) | 2 (12.5) | 3.1 (0.3-38) | 0.368 |
| Dyspnea | 0 (0.0) | 1 (6.2) | --- | 0.994 |
| Fatigue | 1 (4.3) | 0 (0.0) | --- | 0.995 |
| Diarrhea | 2 (8.7) | 2 (12.5) | 1.5 (0.2-11.9) | 0.702 |
| Myalgia | 2 (8.7) | 1 (6.2) | 0.7 (0.1-8.4) | 0.779 |
| Headache | 3 (13.0) | 0 (0.0) | --- | 0.994 |
| Dizziness | 0 (0.0) | 2 (12.5) | --- | 0.992 |
| Chill and rigor | 3 (13.0) | 3 (18.8) | 1.5 (0.3-8.8) | 0.629 |
| Nausea or vomiting | 1 (4.3) | 1 (6.2) | 1.5 (0.1-25.3) | 0.792 |
| **Co–morbidities – no. (%)** |  |  |  |  |
| Hypertension | 1 (4.3) | 1 (6.2) | 1.5 (0.1-25.3) | 0.792 |
| Diabetes mellitus | 1 (4.3) | 1 (6.2) | 1.5 (0.1-25.3) | 0.792 |
| Hyperlipidemia | 0 (0.0) | 1 (6.2) | --- | 0.994 |
| Liver disease | 0 (0.0) | 3 (18.8) | --- | 0.994 |
| Chronic pulmonary disease | 1 (4.3) | 0 (0.0) | --- | 0.995 |
| Renal disease | 0 (0.0) | 1 (6.2) | --- | 0.994 |
| Coronary artery disease | 1 (4.3) | 1 (6.2) | 1.5 (0.1-25.3) | 0.792 |
| **Laboratory result (within 48 hours of admission)** |  |  |  |  |
| Median white cell count (IQR) – x109 | 4.70 [3.94,6.47] | 3.88 [3.15,5.36] | 0.7 (0.4-1.1) | 0.131 |
| Median neutrophil count (IQR) – x109 | 2.73 [2.08,4.05] | 2.23 [1.54,2.56] | 0.7 (0.4-1.2) | 0.167 |
| Median lymphocyte count (IQR) – x109 | 1.32 [0.96,1.70] | 1.30 [0.84,1.75] | 1.2 (0.3-4.4) | 0.748 |
| Median Alanine aminotransferase (IQR) – U/L** | 16.00 [13.00,31.00] | 20.00 [15.85,29.12] | 1.0 (0.99-1.0) | 0.384 |
| Median Aspartate transaminase (IQR) – U/L** | 22.00 [16.00,28.00] | 25.50 [22.00,30.92] | 1.0 (0.99-1.1) | 0.358 |
| Median Creatinine (IQR) – μ mol/L** | 59.00 [51.50,71.50] | 61.95 [50.85,68.00] | 1.0 (0.99-1.0) | 0.540 |
| Median C–reactive protein (IQR) – mg/L** | 4.50 [1.73,16.25] | 9.88 [3.25,16.55] | 1.0 (0.99-1.1) | 0.378 |
| *For group comparison between patients required and never used O2, logistic regression was used. | | | | |
