# Supplementary material for: SARS-CoV-2 non-structural protein 6 triggers NLRP3-dependent pyroptosis by targeting ATP6AP1
Source: Cell Death Differ. 2022 Jan 8;29(6):1240–54. doi: 10.1038/s41418-021-00916-7 (PMC9177730; doi:10.1038/s41418-021-00916-7)
Supplement: Supplementary file 2 — Supplementary Figure Legends [file 41418_2021_916_MOESM2_ESM.docx]

**SARS-CoV-2 Non-Structural Protein 6 Triggers NLRP3-dependent Pyroptosis by Targeting ATP6AP1**

**Supplementary Figure Legends**

**Figure S1. Progress of and treatment provided to the 39 COVID-19 patients used for determining the prognostic significance of serum M65 level.** Day of admission was set as day 0.

**Figure S2.** **Gene expression of SARS-CoV-2 in terms of Transcripts Per Million (TPM) in four COVID-19 patients.**

**Figure S3. Knockdown of GSDMD attenuated NSP6-induced cell death.** (**A-D**) A549 and BEAS2B cells were transfected with scrambled control siRNA or GSDMD-specific siRNA (RiboBio, Guangzhou, China) for 24 h followed by transfection with control empty vector or NSP6-encoding plasmid for another 30 h. (**A**) Cell death monitored by PI staining and active CASP1 identified by FLICA probe were analyzed by flow cytometry. (**B-D**) Quantification data of CASP1^+^ (**B**), PI^+^ (**C**) and CASP1^+^PI^+^ (**D**) cells are displayed as percentages. Significance was assessed using two-way ANOVA with Sidak’s multiple comparison test. All the quantitative data are presented as means ± SD. ***P* < 0.01; ***, *p* < 0.001.

**Figure S4. The effect of NSP6 on caspase-3 cleavage.** Calu-3, A549 and BEAS2B cells were transfected with scrambled control siRNA or three different NLRP3-specific siRNAs, two different CASP1-specific siRNAs and one ASC-specific siRNA for 24 h followed by transfection with control empty vector or NSP6-encoding plasmid for another 24 h. Whole cell lysates were examined for inactive/full-length and active forms of CASP3 (p17).

**Figure S5. 1α,25-Dihydroxyvitamin D_3_, metformin and polydatin restored autophagic flux and attenuated NSP6-induced inflammasome activation and pyroptosis.** (**A-B**) A549 cells were pretreated without or with 1α,25-Dihydroxyvitamin D_3_ (1,25D_3_; 200 nM), metformin (100 μM) or polydatin (25 μM) for 16 h and then co-transfected with mCherry-GFP-LC3 plasmid and control empty vector or NSP6-encoding plasmid for 24 h. Indicated drugs were added to the culture again 8 h after transfection until the cells were harvested. (**A**) Representative fluorescence images of mCherry-GFP-LC3 fusion protein (GFP, green; mCherry, red) and His-tagged NSP6 (magenta) expression. Nuclei were stained with DAPI (blue). Scale bar 10 μm. (**B**) Quantification of colocalization coefficient is displayed as the percentage of punctate mCherry signals that were positive for GFP signals. Twenty cells from each group were randomly selected for statistical analysis. (**C-D**) A549 cells were pretreated without or with 1,25D_3_ (200 nM) and then transfected with control empty vector or NSP6-encoding plasmid for 24 h. 1,25D3 (200 nM) was added to the culture again 8 h after transfection until examination. (**C**) Representative fluorescence images of acridine orange (AO) staining. Scale bar 50 μm. (**D**) AO^+^ cells were quantitated by flow cytometry and are shown as percentage. The dashed line indicates the gate between AO^-^ and AO^+^ cells. (**E-J**) A549, BEAS2B and 16HBE cells were pretreated without or with 1,25D3 (200 nM), metformin (100 μM) or polydatin (25 μM) for 16 h followed by transfection with control empty vector or NSP6-encoding plasmid for 24 h. Indicated drugs were added to the culture again 8 h after transfection until the cells were harvested. Chloroquine (CQ) at the indicated concentrations was added to cultures 1 h after transfection until examination. (**E**) Whole cell lysates were examined for His-tagged NSP6, LC3B, SQSTM1/p62, IL-18, inactive/full-length and active forms of CASP1 (p20), GSDMD (p30 NT fragment) as well as IL-1β (p17). (**F**) Quantification of IL-1β in culture supernatants shows that 1,25D3, metformin and polydatin attenuated whereas CQ exacerbated NSP6-induced IL-1β secretion. (**I**) Cell death monitored by PI staining and active CASP1 identified by FLICA probe were analyzed by flow cytometry. (**H-J**) Quantification data of CASP1^+^ (**H**), PI^+^ (**I**) and CASP1^+^PI^+^ (**J**) cells are displayed as percentages. Significance was assessed using one-way ANOVA with Tukey’s multiple comparison test (**D**) or two-way ANOVA with Sidak’s multiple comparison test (**B, F, H-J**). All the quantitative data are presented as means ± SD. ***P* < 0.01; ***, *p* < 0.001; ^†^, *p* < 0.05; ^††^, *p* < 0.01; ^†††^, *p* < 0.001 when compared to untreated NSP6 group.

**Figure S6. NSP6-triggered reactive oxygen species (ROS) production was attenuated by 1α,25-dihydroxyvitamin D3, metformin and polydatin in airway epithelial cells.** 16HBE and BEAS2B cells were pretreated without or with 1α,25-Dihydroxyvitamin D_3_ (1,25D_3_; 200 nM), metformin (100 μM) or polydatin (25 μM) for 16 h then transfected with control empty vector or His-tagged NSP6-encoding plasmid for 48 h. Indicated drugs were added to the cultures again 8 h post transfection until further examination. ROS probe H_2_DCFDA (10 μM) was added to cells under experimental conditions for 1 h in darkness to determine the intracellular ROS levels. DCFH fluorescence intensities (mean) were analyzed using flow cytometry (n = 3). ***, *p* < 0.001; ^†††^, *p* < 0.001 when compared to untreated NSP6 group.

**Figure S7. SARS-CoV-2 triggered pyroptosis was attenuated by NSP6 knockdown and 1α,25-Dihydroxyvitamin D_3_. (A)** A549 cells were transfected with scrambled control siRNA or three different sets of siRNA targeting NSP6 (50 nM, 20 h), followed by inoculation with SARS-CoV-2 (MOI = 1) for 48 h. Whole-cell lysates were examined for LC3B, SQSTM1/p62, NLRP3, as well as inactive/full-length and active forms of CASP1 and GSDMD. (B) A549 cells were pretreated without or with 1α,25-dihydroxyvitamin D_3_ (1,25D_3_; 200 nM) for 48 h, followed by inoculation with SARS-CoV-2 (MOI = 1). One hour post inoculation, the culture medium was removed and the cells were washed twice with PBS, then cultured in fresh medium supplied without or with 1,25D_3_ (200 nM) for 48 h. Whole-cell lysates were examined for the inactive/full-length and active forms of CASP1 and GSDMD.β-Actin was used as a loading control.
