# Supplementary figures and images for: SARS-CoV-2 non-structural protein 6 triggers NLRP3-dependent pyroptosis by targeting ATP6AP1
Source: Cell Death Differ. 2022 Jan 8;29(6):1240–54. doi: 10.1038/s41418-021-00916-7 (PMC9177730; doi:10.1038/s41418-021-00916-7)

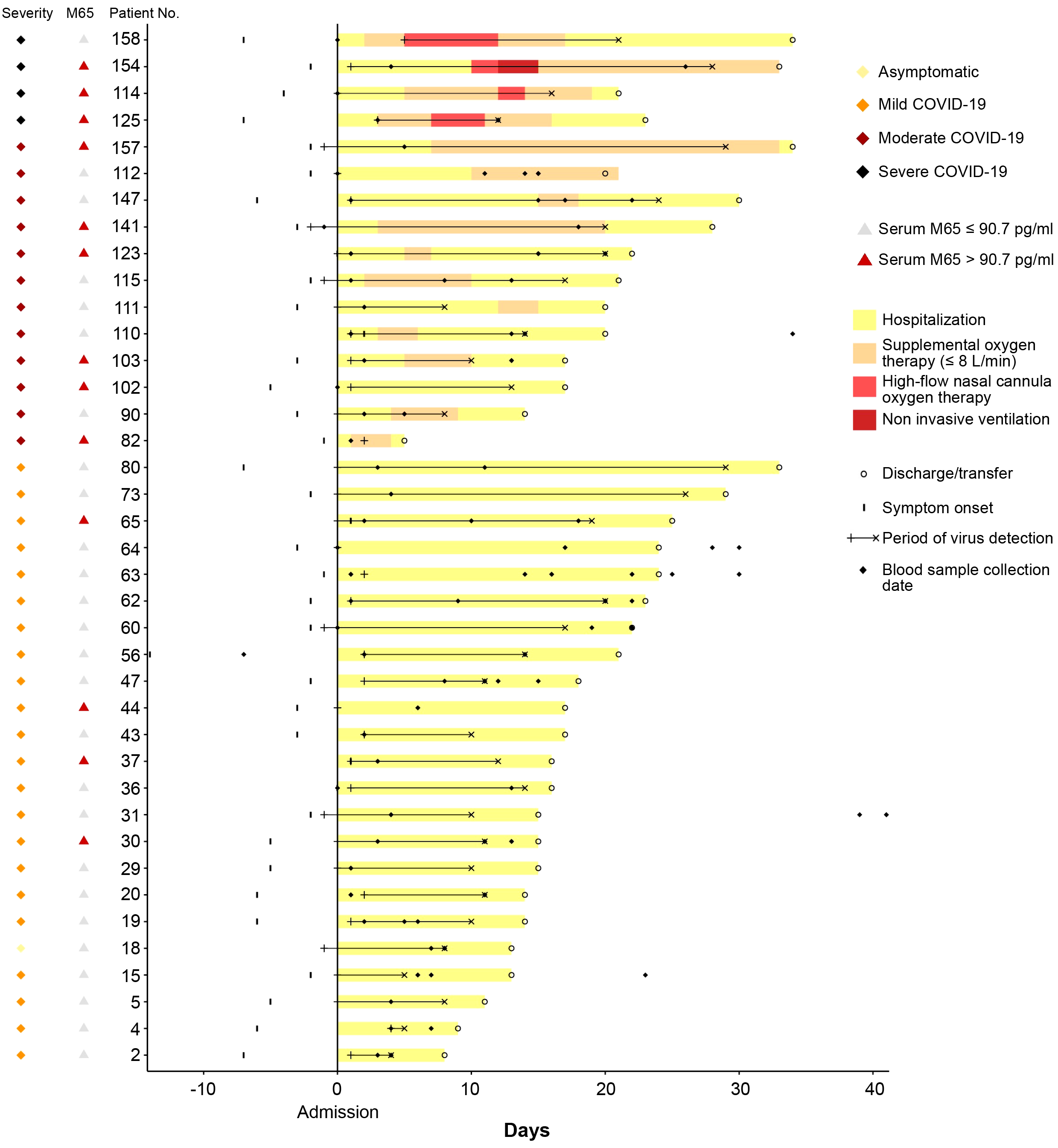

Supplement: Supplementary file 3 — Supplementary Figure S1 [file 41418_2021_916_MOESM3_ESM.tif]

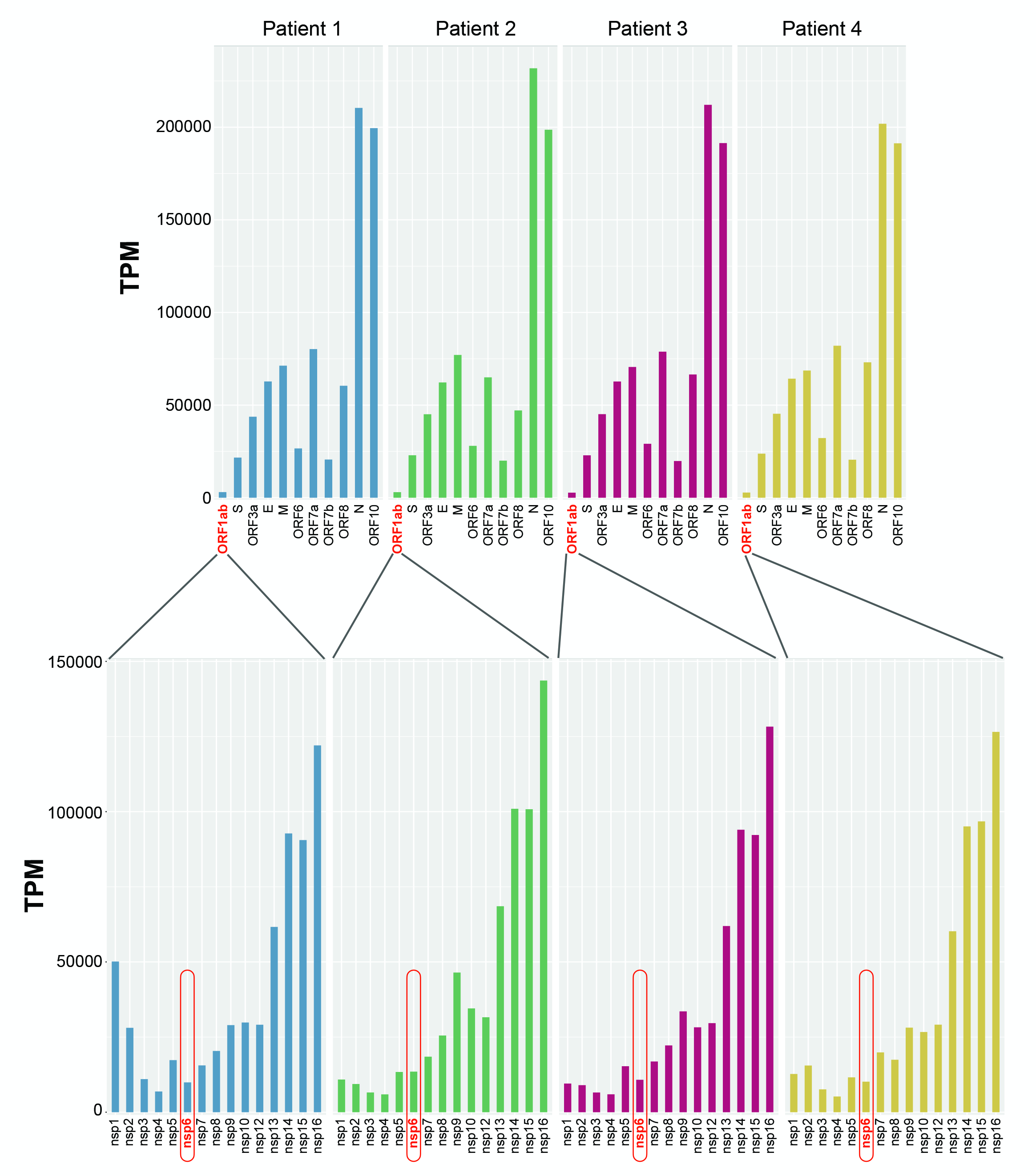

Supplement: Supplementary file 4 — Supplementary Figure S2 [file 41418_2021_916_MOESM4_ESM.tif]

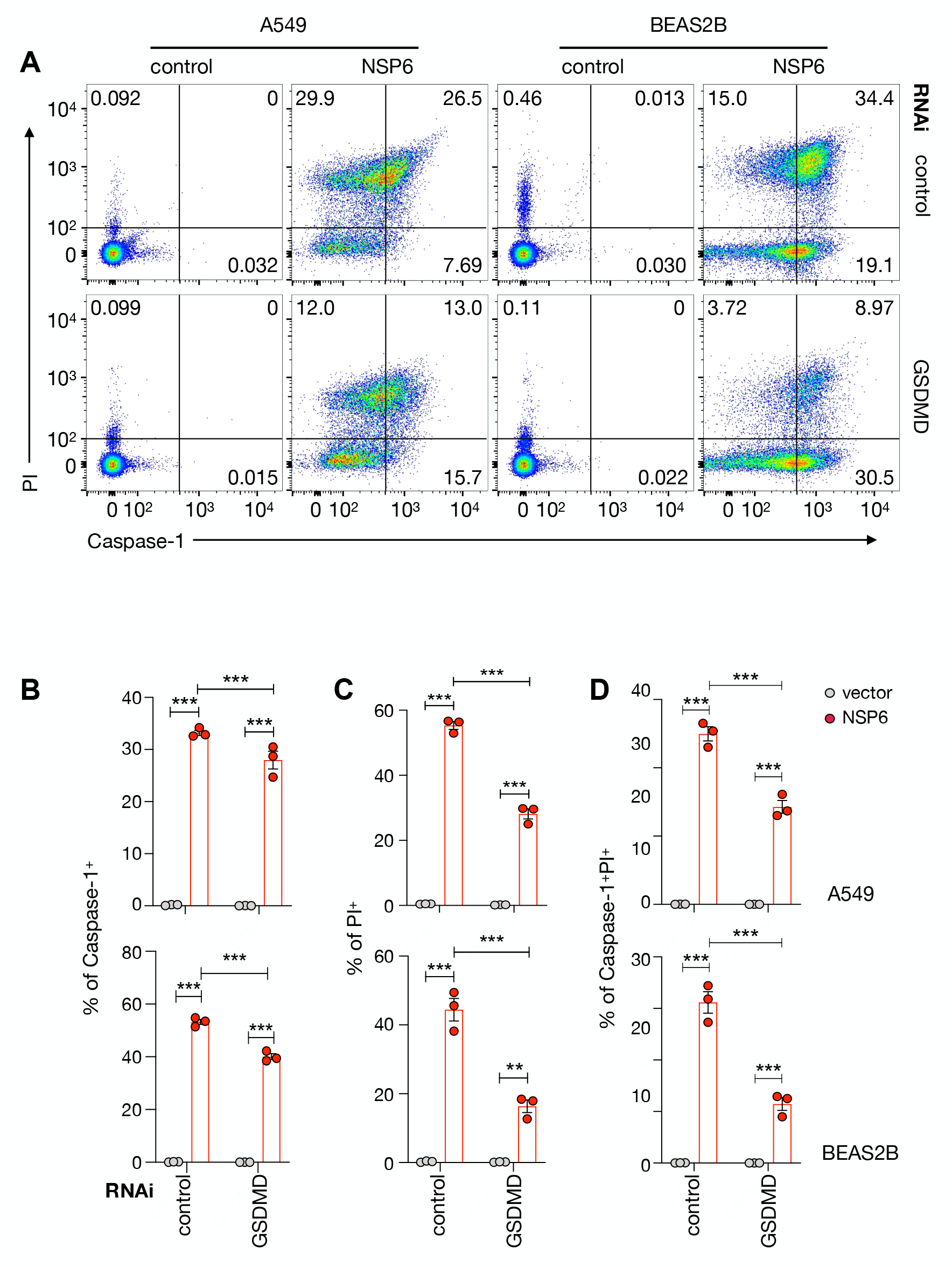

Supplement: Supplementary file 5 — Supplementary Figure S3 [file 41418_2021_916_MOESM5_ESM.tif]

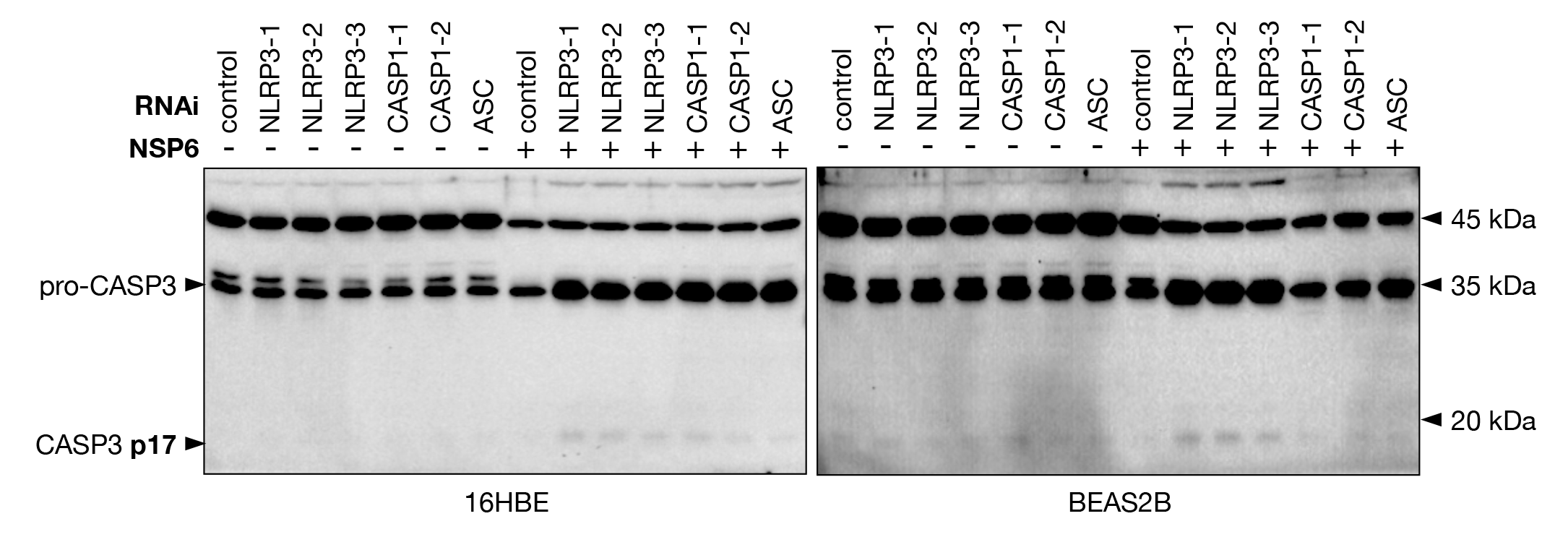

Supplement: Supplementary file 6 — Supplementary Figure S4 [file 41418_2021_916_MOESM6_ESM.tif]

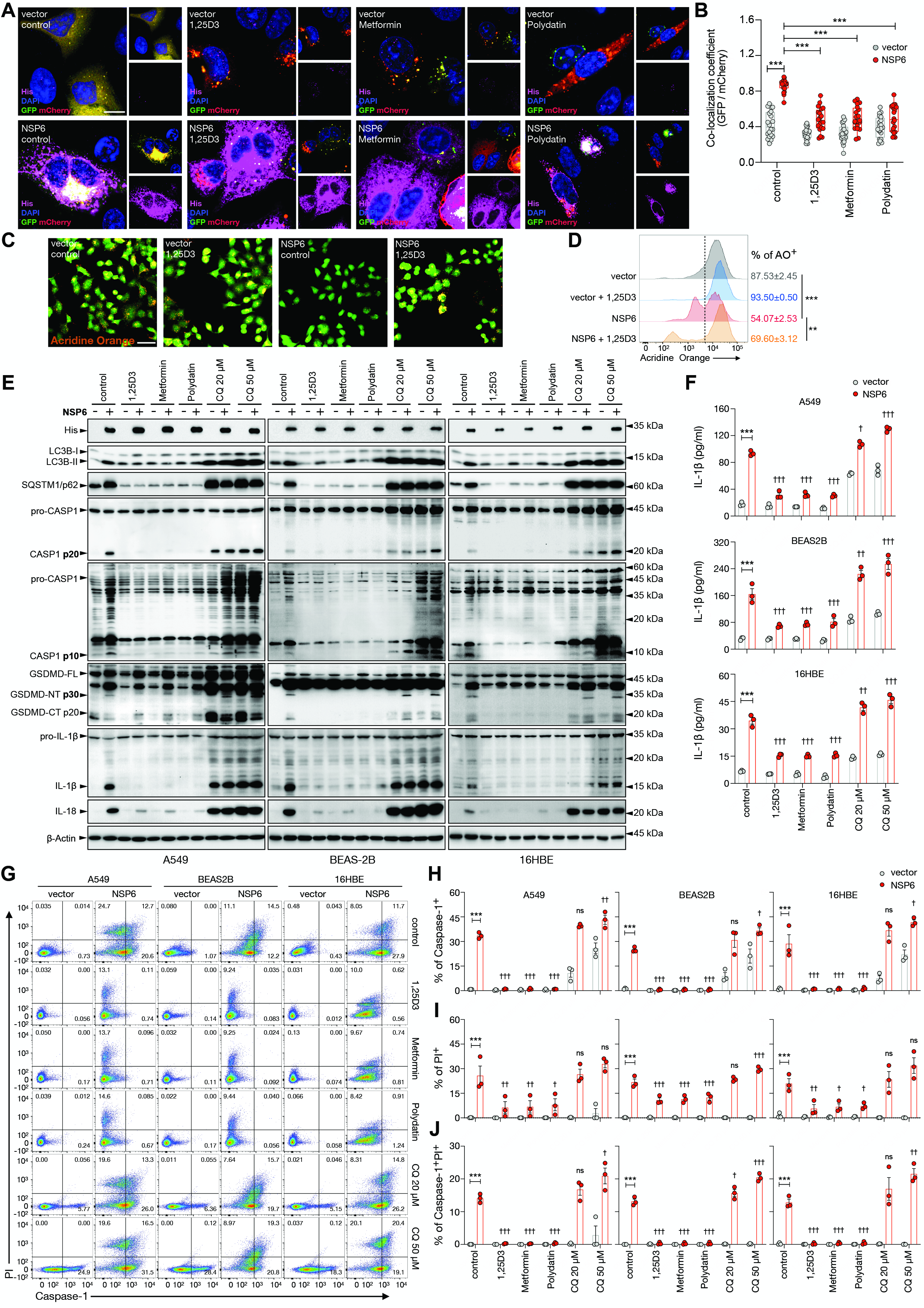

Supplement: Supplementary file 7 — Supplementary Figure S5 [file 41418_2021_916_MOESM7_ESM.tif]

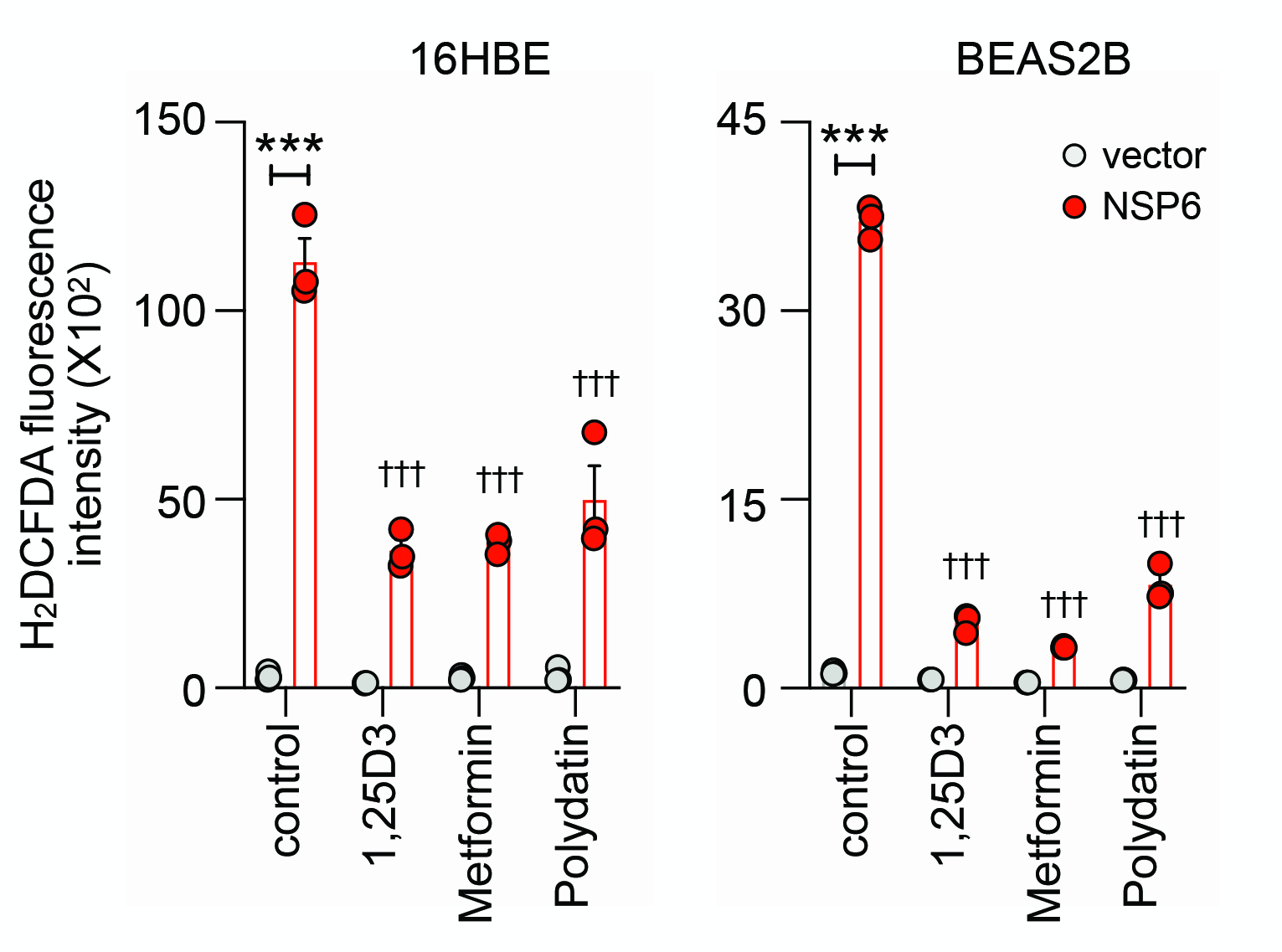

Supplement: Supplementary file 8 — Supplementary Figure S6 [file 41418_2021_916_MOESM8_ESM.tif]

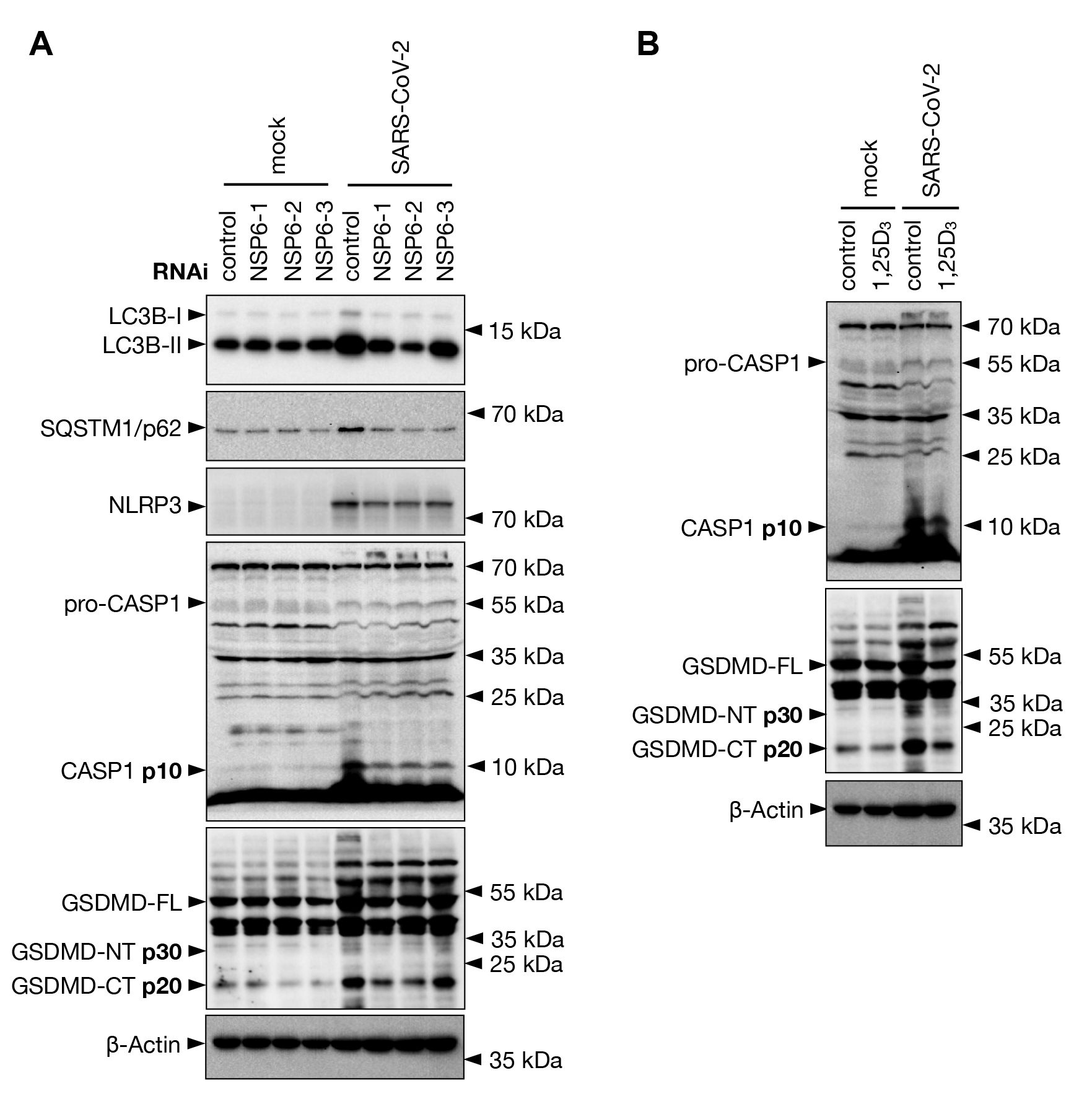

Supplement: Supplementary file 9 — Supplementary Figure S7 [file 41418_2021_916_MOESM9_ESM.tif]
