# Supplementary material for: SARS-CoV-2 non-structural protein 6 triggers NLRP3-dependent pyroptosis by targeting ATP6AP1
Source: Cell Death Differ. 2022 Jan 8;29(6):1240–54. doi: 10.1038/s41418-021-00916-7 (PMC9177730; doi:10.1038/s41418-021-00916-7)
Supplement: Supplementary file 11 — Change of authorship request form [file 41418_2021_916_MOESM11_ESM.pdf]

Change of authorship request form - Journals (pre-acceptance)

SPRINGER NATURE

Important information. Please read.

- This form should be used by authors to request any change in authorship (adding/deleting authors) including changes in corresponding authors. This form should not be used for name changes. Please fully complete all sections. Use black ink and block capitals and provide each author's full name with the given name first followed by the family name.

- By signing this declaration, all authors guarantee that the order of the authors are in accordance with their scientific contribution, if applicable as different conventions apply per discipline, and that only authors have been added who made a meaningful contribution to the work.

- Please note, in author collaborations where there is formal agreement for representing the collaboration, it is sufficient for the representative or legal guarantor (usually the corresponding author) to complete and sign the Authorship Change Form on behalf of all authors, **next to the added/removed author(s)**. **(Complete Section 3, followed by Section 6.)**
  In author collaborations where there is no formal agreement for representing the collaboration and **there are more than 10 authors**, one may sign for all, provided the signer appends correspondence that attests that each of the authors have agreed to the change **and the added/removed authors sign the form**. **(Complete Section 3, followed by Section 6.)**

- Please note, we cannot investigate or mediate any authorship disputes. If you are unable to obtain agreement from all authors (including those who you wish to be removed) you must refer the matter to your institution(s) for investigation. Please inform us if you need to do this.

- If you are not able to return a fully completed form within **30 days** of the date that it was sent to the author requesting the change, we may have to withdraw your manuscript. We cannot publish manuscripts where authorship has not been agreed by all authors (including those who have been removed).

- Incomplete forms will be rejected.

- Please return/upload this form, fully completed, to the Journals Editorial Office. The Journal and/or Publisher will consider the information you have provided to decide whether to approve the proposed change in authorship. We may decide to contact your institution for more information or undertake a further investigation, if appropriate, before making a final decision.

SPRINGER NATURE Change of authorship request form - Journals (pre-acceptance)

Section 1: Please provide the current title of manuscript

Manuscript ID no.: CDD-21-1422R

Title: SARS-CoV-2 Non-Structural Protein 6 Triggers NLRP3-dependent Pyroptosis by Targeting ATP6AP1

Section 2: Please provide the previous authorship, in the order shown on the manuscript before the changes were introduced. Please indicate the corresponding author by adding (CA) behind the name.

	First name(s)	Family name	ORCID or SCOPUS id, if available
1st author	Xiao	Sun	
2nd author	Yingzhi	Liu	
3rd author	Ziheng	Huang	
4th author	Wenye	Xu	
5th author	Wei	Hu	
6th author	Hung	Chan	
7th author	Judeng	Zeng	
8th author	Xiaodong	Liu	
9th author	Huarong	Chen	37119896400
10th author	Jun	Yu	35351306800

Please use an additional sheet if there are more than 10 authors.

SPRINGER NATURE Springer Nature is one of the world's leading global research, educational and professional publishers, created in May 2015 through the combination of Nature Publishing Group, Palgrave Macmillan, Macmillan Education and Springer Science+Business Media.

Title: SARS-CoV-2 Non-Structural Protein 6 Triggers NLRP3-dependent Pyroptosis by Targeting ATP6AP1
Manuscript ID no.: CDD-21-1422R

	First name(s)	Family name	ORCID or SCOPUS id, if available
11th author	Francis Ka Leung	Chan	0000-0001-7388-2436
12th author	Siew Chien	Ng	0000-0002-6850-4454
13th author	Sunny Hei	Wong	0000-0002-3354-9310
14th author	Maggie Haitian	Wang	55596673500
15th author	Tony	Gin	0000-0001-7283-6761
16th author	Gavin Matthew	Joynt	7005588815
17th author	David Shu Cheong	Hui	0000-0003-4382-2445
18th author	Xuan	Zou	
19th author	Yuelong	Shu	
20th author	Shisong	Fang	7402422510
21st author	Huanle	Luo	56486560600
22nd author	Matthew Tak Vai	Chan	0000-0002-3574-7855
23rd author	Christopher Hon Ki	Cheng	57220698091
24th author	Lin	Zhang	0000-0003-1634-3780
25th author	William Ka Kei	Wu	0000-0002-5662-5240

SPRINGER NATURE

Change of authorship request form - Journals (pre-acceptance)

Section 3: Please provide a justification for change. Please use this section to explain your reasons for changing the authorship of your manuscript, e.g. what necessitated the change in authorship? Please refer to the (journal) policy pages for more information about authorship. Please explain why omitted authors were not originally included and/or why authors were removed on the submitted manuscript.

1. Lina Yi, Zhe Liu and Jing Lu are added as new authors. These 3 authors contributed to the execution of live SARS-CoV-2 infection experiments.

2. Jing Lu is a new co-corresponding author. He contributed to the coordination of live SARS-CoV-2 infection experiments.

3. Christopher Hon Ki Cheng can no longer serve as co-corresponding author due to his untimely demise after the initial submission (https://www2.sbs.cuhk.edu.hk/en-gb/news-and-events/news/2021-news/1305-in-memoriam-prof-cheng-hon-ki-christopher)

Section 4: Proposed new authorship. Please provide your new authorship list in the order you would like it to appear on the manuscript. Please indicate the corresponding author by adding (CA) behind the name. If the Corresponding Author has changed, please indicate the reason under section 3.

	First name(s)	Family name (this name will appear in full on the final publication and will be searchable in various abstract and indexing databases)	Affiliated institute	E-mail address
1st author	Xiao	Sun	The Chinese University of Hong Kong	sunxiaosara@gmail.com
2nd author	Yingzhi	Liu	The Chinese University of Hong Kong	wing_lau@outlook.com
3rd author	Ziheng	Huang	The Chinese University of Hong Kong	zhhuang@link.cuhk.edu.hk
4th author	Wenye	Xu	The Chinese University of Hong Kong	xuwenxiaoye@163.com
5th author	Wei	Hu	Southern Medical University	huwei1683013@163.com
6th author	Lina	Yi	Guangdong Provincial Institution of Public Health and Guangdong Provincial Center for Disease Control and Prevention	linayi2009@live.cn
7th author	Zhe	Liu	Guangdong Provincial Institution of Public Health and Guangdong Provincial Center for Disease Control and Prevention	lzhwin@foxmail.com
8th author	Hung	Chan	The Chinese University of Hong Kong	marcuschan64@gmail.com
9th author	Judeng	Zeng	The Chinese University of Hong Kong	zjdseed@163.com
10th author	Xiaodong	Liu	The Chinese University of Hong Kong	xdliu@cuhk.edu.hk

Please use an additional sheet if there are more than 10 authors.

SPRINGER NATURE Springer Nature is one of the world's leading global research, educational and professional publishers, created in May 2015 through the combination of Nature Publishing Group, Palgrave Macmillan, Macmillan Education and Springer Science+Business Media.

Title: SARS-CoV-2 Non-Structural Protein 6 Triggers NLRP3-dependent Pyroptosis by Targeting ATP6AP1
Manuscript ID no.: CDD-21-1422R

	First name(s)	Family name (this name will appear in full on the final publication and will be searchable in various abstract and indexing databases)	Affiliated institute	E-mail address
11th author	Huarong	Chen	The Chinese University of Hong Kong	hchen2@cuhk.edu.hk
12th author	Jun	Yu	The Chinese University of Hong Kong	junyu@cuhk.edu.hk
13th author	Francis Ka Leung	Chan	The Chinese University of Hong Kong	fklchan@cuhk.edu.hk
14th author	Siew Chien	Ng	The Chinese University of Hong Kong	siewchienng@cuhk.edu.hk
15th author	Sunny Hei	Wong	The Chinese University of Hong Kong	wonghei@cuhk.edu.hk
16th author	Maggie Haitian	Wang	The Chinese University of Hong Kong	maggiew@cuhk.edu.hk
17th author	Tony	Gin	The Chinese University of Hong Kong	tgin@cuhk.edu.hk
18th author	Gavin Matthew	Joynt	The Chinese University of Hong Kong	gavinmjoynt@cuhk.edu.hk
19th author	David Shu Cheong	Hui	The Chinese University of Hong Kong	dschui@cuhk.edu.hk
20th author	Xuan	Zou	Shenzhen Center for Disease Control and Prevention	914494557@qq.com
21st author	Yuelong	Shu	Sun Yat-sen University	shuylong@mail.sysu.edu.cn
22nd author	Christopher Hon Ki	Cheng	The Chinese University of Hong Kong	chkcheng@cuhk.edu.hk

Title: SARS-CoV-2 Non-Structural Protein 6 Triggers NLRP3-dependent Pyroptosis by Targeting ATP6AP1
Manuscript ID no.: CDD-21-1422R

23rd author	Shisong	Fang	Shenzhen Center for Disease Control and Prevention	szcdcssf@aliyun.com
24th author	Huanle	Luo	Sun Yat-sen University	luohle@mail.sysu.edu.cn
25th author	Jing	Lu	Guangdong Provincial Institution of Public Health and Guangdong Provincial Center for Disease Control and Prevention	jimlu0331@163.com
26th author	Matthew Tak Vai	Chan	The Chinese University of Hong Kong	mtvchan@cuhk.edu.hk
27th author	Lin	Zhang	The Chinese University of Hong Kong	linzhang@cuhk.edu.hk
28th author	William Ka Kei	Wu	The Chinese University of Hong Kong	wukakei@cuhk.edu.hk

SPRINGER NATURE

Change of authorship request form - Journals (pre-acceptance)

Section 5: Author contribution, Acknowledgement and Disclosures. Please use this section to provide a new disclosure statement and, if appropriate, acknowledge any contributors who have been removed as authors and ensure you state what contribution any new authors made (if applicable per the journal or book (series) policy). Please ensure these are updated in your manuscript - after approval of the change(s) - as our production department will not transfer the information in this form to your manuscript.

New acknowledgements:

Not applicable

New Disclosures (financial and non-financial interests, funding):

Not applicable

New Author Contributions statement (if applicable per the journal policy):

Conceptualization: WKKW
Methodology: WKKW
Formal analysis: XS, JZ, XL
Investigation: XS, JL, ZL, LNY, YL, ZH, WX
Resources: XZ, YS, SF, HL, JL
Writing – Original Draft: XS
Writing – Review: WKKW
Writing – Editing: LZ, JY, FKLC, SCN, SHW, TG, GMJ, DSCH
Supervision: WKKW, CHKC, MTVC, LZ

State 'Not applicable' if there are no new authors.

SPRINGER NATURE Springer Nature is one of the world's leading global research, educational and professional publishers, created in May 2015 through the combination of Nature Publishing Group, Palgrave Macmillan, Macmillan Education and Springer Science+Business Media.

SPRINGER NATURE

Change of authorship request form - Journals (pre-acceptance)

Section 6: Declaration of agreement. *All* authors, unchanged, new and removed *must* sign this declaration.

(NB: Please print the form, (docu)-sign and return/upload a scanned copy. Please note that signatures that have been inserted as an image file are acceptable as long as it is handwritten. Typed names in the signature box are unacceptable.) *** Please delete as appropriate. Delete all of the bold if you were on the original authorship list and are remaining as an author.**

	First name	Family name		Signature	Date
1st author	Xiao	Sun	I agree to the proposed new authorship shown in section 4 /~~**and the addition/removal*of my name to the authorship list**~~/and the proposed change in corresponding author	Sun Xiao	**03 Nov 2021**
2nd author	Yingzhi	Liu	I agree to the proposed new authorship shown in section 4 /~~**and the addition/removal*of my name to the authorship list**~~ /and the proposed change in corresponding author	Liu Yingzhi.	**03 Nov 2021**
3rd author	Ziheng	Huang	I agree to the proposed new authorship shown in section 4 /~~**and the addition/removal*of my name to the authorship list**~~ /and the proposed change in corresponding author	Huang Ziheng	**03 Nov 2021**
4th authors	Wenye	Xu	I agree to the proposed new authorship shown in section 4 /~~**and the addition/removal*of my name to the authorship list**~~ /and the proposed change in corresponding author	XU Wenye	**03 Nov 2021**
5th author	Wei	Hu	I agree to the proposed new authorship shown in section 4 /~~**and the addition/removal*of my name to the authorship list**~~ /and the proposed change in corresponding author	HuWei	**02 Nov 2021**
6th author	Lina	Yi	I agree to the proposed new authorship shown in section 4 /**and the addition/~~removal~~*of my name to the authorship list** /and the proposed change in corresponding author	Lina Yi	**03 Nov 2021**
7th author	Zhe	Liu	I agree to the proposed new authorship shown in section 4 /**and the addition/~~removal~~*of my name to the authorship list** /and the proposed change in corresponding author	Zhe Liu	**03 Nov 2021**

SPRINGER NATURE Springer Nature is one of the world's leading global research, educational and professional publishers, created in May 2015 through the combination of Nature Publishing Group, Palgrave Macmillan, Macmillan Education and Springer Science+Business Media.

SPRINGER NATURE Change of authorship request form - Journals (pre-acceptance)

	First name	Family name		Signature	Date
8th author	Hung	Chan	I agree to the proposed new authorship shown in section 4 /~~and the addition/removal*of my name to the authorship list~~ /and the proposed change in corresponding author	Hung	3 Nov 2021
9th author	Judeng	Zeng	I agree to the proposed new authorship shown in section 4 /~~and the addition/removal*of my name to the authorship list~~ /and the proposed change in corresponding author	[illegible]	3 Nov 2021
10th author	Xiaodong	Liu	I agree to the proposed new authorship shown in section 4 /~~and the addition/removal*of my name to the authorship list~~ /and the proposed change in corresponding author	liuxiaodong	02/11/2021

Please use an additional sheet if there are more than 10 authors.

In case of author collaborations with formal agreement:

	Name of consortium/consortia	First name	Family name		Signature	Date
Representative/legal guarantor				I agree to the proposed new authorship shown in section 4 /**and the addition/removal*of my name to the authorship list** /and the proposed change in corresponding author		

Both added/removed authors should complete the information in the first table under Section 6.

---- End of form ----

SPRINGER NATURE Springer Nature is one of the world's leading global research, educational and professional publishers, created in May 2015 through the combination of Nature Publishing Group, Palgrave Macmillan, Macmillan Education and Springer Science+Business Media.

Title: SARS-CoV-2 Non-Structural Protein 6 Triggers NLRP3-dependent Pyroptosis by Targeting ATP6AP1
Manuscript ID no.: CDD-21-1422R

	First name	Family name		Signature	Date
11th author	Huarong	Chen	I agree to the proposed new authorship shown in section 4 /~~and the addition/removal*of my name to the authorship list /~~and the proposed change in corresponding author		2 Nov 2021
12th author	Jun	Yu	I agree to the proposed new authorship shown in section 4 /~~and the addition/removal*of my name to the authorship list /~~and the proposed change in corresponding author		2 Nov 2021
13th author	Francis Ka Leung	Chan	I agree to the proposed new authorship shown in section 4 /~~and the addition/removal*of my name to the authorship list /~~and the proposed change in corresponding author		3 Nov 2021
14th author	Siew Chien	Ng	I agree to the proposed new authorship shown in section 4 /~~and the addition/removal*of my name to the authorship list /~~and the proposed change in corresponding author		3 Nov 2021
15th author	Sunny Hei	Wong	I agree to the proposed new authorship shown in section 4 /~~and the addition/removal*of my name to the authorship list /~~and the proposed change in corresponding author		3 Nov 2021
16th author	Maggie Haitian	Wang	I agree to the proposed new authorship shown in section 4 /~~and the addition/removal*of my name to the authorship list /~~and the proposed change in corresponding author		3 Nov 2021
17th author	Tony	Gin	I agree to the proposed new authorship shown in section 4 /~~and the addition/removal*of my name to the~~		4 Nov 2021

Title: SARS-CoV-2 Non-Structural Protein 6 Triggers NLRP3-dependent Pyroptosis by Targeting ATP6AP1
Manuscript ID no.: CDD-21-1422R

			~~authorship list /~~and the proposed change in corresponding author		
18th author	Gavin Matthew	Joynt	I agree to the proposed new authorship shown in section 4 /~~and the addition/removal*of my name to the authorship list /~~and the proposed change in corresponding author		3 Nov 2021
19th author	David Shu Cheong	Hui	I agree to the proposed new authorship shown in section 4 /~~and the addition/removal*of my name to the authorship list /~~and the proposed change in corresponding author		3 Nov 2021
20th author	Xuan	Zhu	I agree to the proposed new authorship shown in section 4 /~~and the addition/removal*of my name to the authorship list /~~and the proposed change in corresponding author		2 Nov 2021
21st author	Yuelong	Shu	I agree to the proposed new authorship shown in section 4 /~~and the addition/removal*of my name to the authorship list /~~and the proposed change in corresponding author		4 Nov 2021
22nd author	Christopher Hon Ki	Cheng	I agree to the proposed new authorship shown in section 4 /~~and the addition/removal*of my name to the authorship list /~~and the proposed change in corresponding author	(signed by William K.K. Wu on behalf of Prof. Christopher Hon Ki Cheng who deceased on 12 July 2021)	2 Nov 2021
23rd author	Shisong	Fang	I agree to the proposed new authorship shown in section 4 /~~and the addition/removal*of my name to the authorship list /~~and the proposed change in corresponding author		2 Nov 2021
24th author	Huanle	Luo	I agree to the proposed new authorship shown in section 4 /~~and the addition/removal*of my name to the~~		2 Nov 2021

			~~authorship list /~~and the proposed change in corresponding author		
25th author	Jing	Lu	I agree to the proposed new authorship shown in section 4 /and the addition/~~removal*~~of my name to the authorship list /and the proposed change in corresponding author	[illegible]	2 Nov 2021
26th author	Matthew Tak Vai	Chan	I agree to the proposed new authorship shown in section 4 /~~and the addition/removal*of my name to the authorship list /~~and the proposed change in corresponding author	[illegible]	2 Nov 2021
27th author	Lin	Zhang	I agree to the proposed new authorship shown in section 4 /~~and the addition/removal*of my name to the authorship list /~~and the proposed change in corresponding author	zhanglin	2 Nov 2021
28th author	William Ka Kei	Wu	I agree to the proposed new authorship shown in section 4 /~~and the addition/removal*of my name to the authorship list /~~and the proposed change in corresponding author	[illegible]	2 Nov 2021
